# *Elsholtzia bodinieri* Vaniot Ameliorated Acute Lung Injury by NQO1, BCL2 and PTGS2 In Silico and In Vitro Analyses

**DOI:** 10.3390/ijms232415651

**Published:** 2022-12-09

**Authors:** Jin Sun, Xiaoqian Jiang, Yuxu Chen, Shancheng Guo, Zhiye Zhao, Jianxin Cao, Yaping Liu, Guiguang Cheng, Ye Li, Lei Tian

**Affiliations:** 1 Faculty of Food Science and Engineering, Kunming University of Science and Technology, Kunming 650500, China; 2 School of Medicine, Kunming University of Science and Technology, Kunming 650500, China

**Keywords:** *Elsholtzia bodinieri* Vaniot, acute lung injury, NQO1, BCL2, PTGS2

## Abstract

Acute lung injury (ALI) is a clinical respiratory disease caused by various factors, which lacks effective pharmacotherapy to reduce the mortality rate. *Elsholtzia bodinieri* Vaniot is an annual herbaceous plant used as a traditional herbal tea and folk medicine. Here we used bioinformatic databases and software to explore and analyze the potential key genes in ALI regulated by *E. bodinieri* Vaniot, including B cell leukemia/lymphoma 2 (*Bcl2*), prostaglandin-endoperoxide synthase 2 (*Ptgs2*) and NAD(P)H dehydrogenase, quinone 1 (*Nqo1*). In an inflammatory cells model, we verified bioinformatics results, and further mechanistic analysis showed that methanol extract of *E. bodinieri* Vaniot (EBE) could alleviate oxidative stress by upregulating the expression of NQO1, suppress pyroptosis by upregulating the expression of BCL2, and attenuate inflammation by downregulating the expression of PTGS2. In sum, our results demonstrated that EBE treatment could alleviate oxidative stress, suppress pyroptosis and attenuate inflammation by regulating NQO1, BCL2 and PTGS2 in a cells model, and *E. bodinieri* Vaniot might be a promising source for functional food or as a therapeutic agent.

## 1. Introduction

ALI is clinical respiratory syndrome caused by various factors, such as COVID-19, toxic inhalation pneumonitis, sepsis, severe trauma, and acute pancreatitis [1,2,3]. The prominent characteristics of ALI involves edema of airways with epithelial sloughing, inflammation of lung and edema with hypoxemia [4]. Existing research showed that the development of ALI was closely related to the inflammatory injury, oxidative stress and immune cell infiltration [5,6,7]. The histopathological lesions of ALI in COVID-19 are of wide concern at the moment. Although various pharmacotherapies are available, the outcome and mortality of ALI patients remains poor [8]. Therefore, there is an urgent need to develop treatments to ameliorate lung inflammation and tissue damage.

*E. bodinieri* Vaniot belongs to the taxonomically diverse group of the family Labiatae, and it is an annual herbaceous plant. The genus *Elsholtzia* consists of at least 33 species, which are widely distributed in East Asia, Africa, and Europe, and some of which have been used as beverages (herbal tea), domestic folk medicine, spices, cosmetics and so on [9]. *E. bodinieri* Vaniot is commonly known as “Dongzisu” in China, which grows in the northwest and southwest districts of China, especially in the Yunnan and Guizhou Provinces [10]. Usually, *E. bodinieri* Vaniot has been used as traditional folk medicine for the treatment of cough, headache, pharyngitis, fever and hepatitis [11]. Previous reports showed that the aerial parts of *E. bodinieri* Vaniot have been isolated clerodane diterpenoid glycosides, flavonoid glycosides, triterpenoid saponins, sesquiterpene glycosides, and phenolic constituents [10,11,12,13,14,15,16].

**Figure 1:**
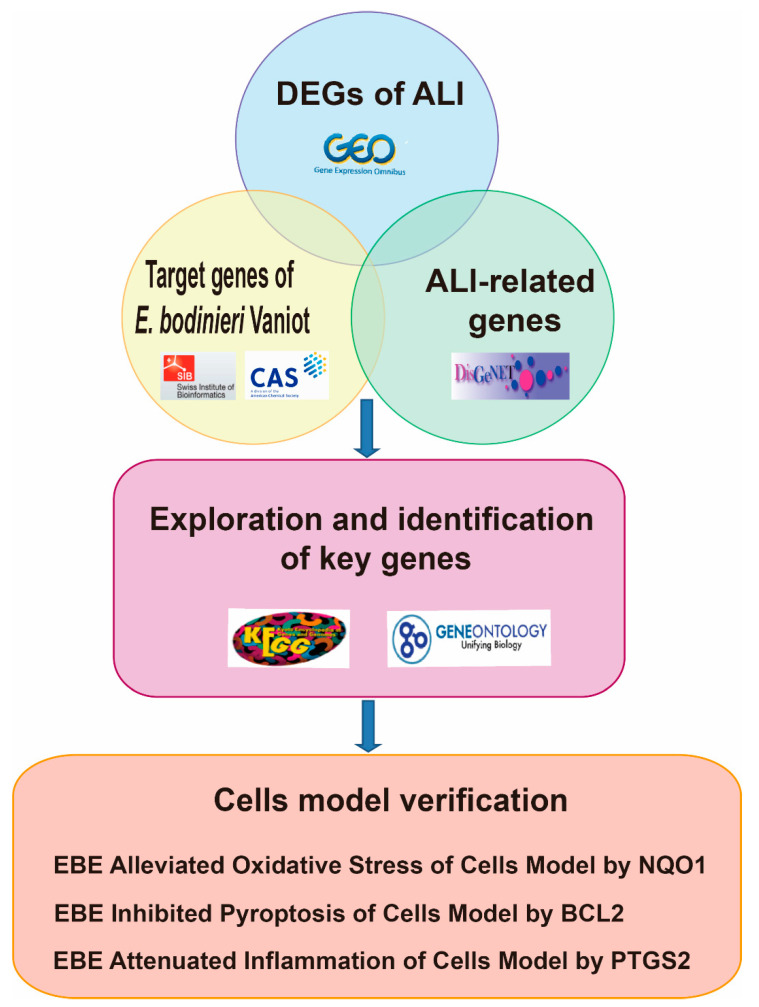
The bioinformatics and experiments workflow of *E. bodinieri* Vaniot ameliorate acute lung injury.

In this work, we use databases and software to explore and analyze potential target genes in ALI, which are regulated by *E. bodinieri* Vaniot. In order to verify the results of the analysis, we investigate the effects of *E. bodinieri* Vaniot on potential target genes, and the mechanism in vitro. The overall design of this study is shown in Figure 1. These findings indicated that *E. bodinieri* Vaniot might be a therapeutic agent for ALI.

## 2. Results

### 2.1. Screening Differentially Expressed Genes and Enrichment Analysis in ALI

**Figure 2:**
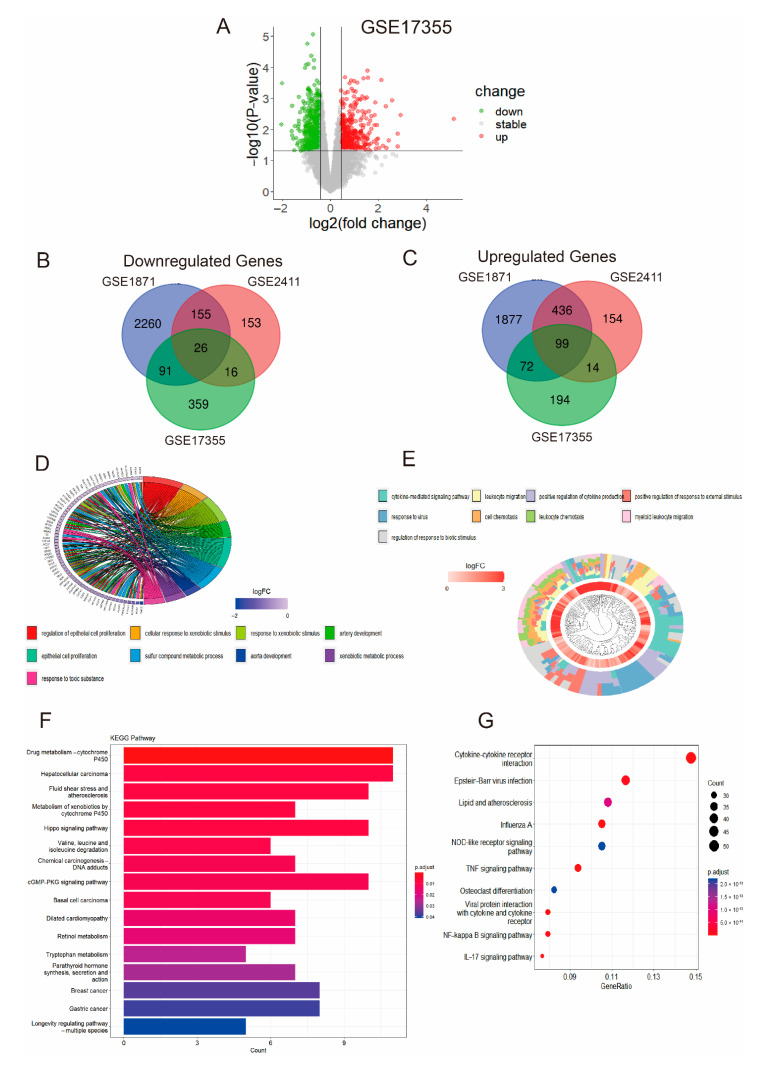
Differentially expressed genes and enrichment analysis in ALI. (**A**) Volcano plot showing DEGs between ALI and normal lung tissue in GSE17355, where red dots indicate upregulated genes, green dots indicate downregulated genes, and gray dots indicate stable genes (corrected *p* values < 0.05). (**B**) Venn diagram of downregulated overlapping genes in the three datasets. (**C**) Venn diagram of upregulated overlapping genes in the three datasets. (**D**) The top nine significant GO terms involving the downregulated overlapping genes. (**E**) The top nine significant GO terms involving the upregulated overlapping genes. (**F**) KEGG pathway analysis of downregulated overlapping genes revealed the top 16 relevant pathways. (**G**) KEGG pathway analysis of upregulated overlapping genes revealed the top ten relevant pathways.

In the GSE17355 dataset, a total of 871 differentially expressed genes (DEGs) in ALI were identified, including 379 upregulated genes and 492 downregulated genes (Figure 2A). In the GSE1871 dataset, a total of 5016 DEGs in ALI were identified, including 2484 upregulated genes and 2532 downregulated genes (Figure S1A). In the GSE2411 dataset, a total of 1053 DEGs in ALI were identified, including 703 upregulated genes and 350 downregulated genes (Figure S1B). We obtained 288 downregulated overlapping genes (Figure 2B) and 621 upregulated overlapping genes (Figure 2C); these genes varied across the three datasets. Next, the downregulated overlapping genes were identified by Gene Ontology (GO) analysis, and the top nine enriched terms were shown (Figure 2D). Meanwhile, upregulated overlapping genes were explored by GO analysis, the top nine enriched terms were also shown (Figure 2E). Finally, the different Kyoto Encyclopedia of Genes and Genomes (KEGG) pathways of downregulated and upregulated overlapping genes were analyzed. The results showed the top 16 KEGG pathways involving downregulated overlapping genes, which included hepatocellular carcinoma, fluid shear stress and atherosclerosis, parathyroid hormone synthesis, secretion and action and so on (Figure 2F). The top ten KEGG pathways involving upregulated overlapping genes included TNF signaling pathway, IL-17 signaling pathway, NF-kappa B signaling pathway and so on (Figure 2G).

### 2.2. Exploration and Identification of Key Genes in ALI Regulated by E. bodinieri Vaniot

**Figure 3:**
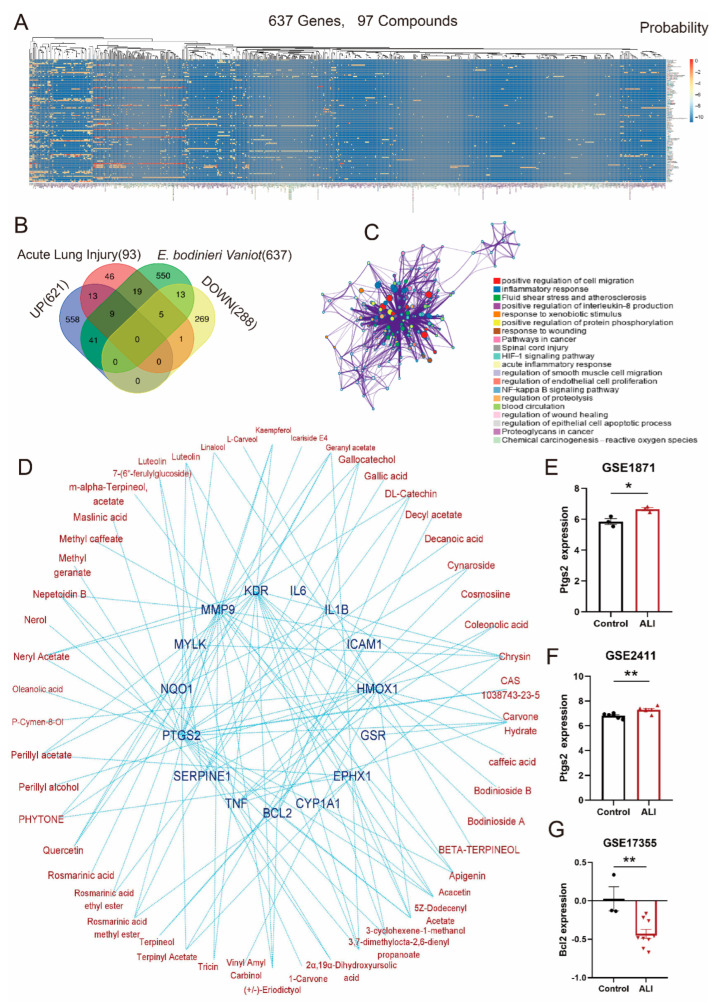
Exploration and identification of key genes in ALI regulated by *E. bodinieri* Vaniot. (**A**) Heat map illustrating the probability between ALI genes and compounds of *E. bodinieri* Vaniot. (**B**) Venn diagram of hub genes in upregulated genes, ALI-related genes, potential target genes of *E. bodinieri* Vaniot, and downregulated genes. (**C**) GO network of hub genes was constructed by Metascape database, and Cytoscape software. (**D**) Key genes-compounds network. The blue nodes represent the key genes, the brown nodes represent the compounds, and the edges represent the interactions between them. (**E**) The expression of *Ptgs2* in GSE1871 dataset, *n* = 3. (**F**) The expression of *Ptgs2* in GSE2411 dataset, *n* = 6. (**G**) The expression of *Bcl2* in GSE17355 dataset, control *n* = 3, ALI *n* = 9. Data are presented as mean ± SEM, * *p* < 0.05, ** *p* < 0.01.

We retrieved 97 compounds of *E. bodinieri* Vaniot from CAS scifinder and PubChem databases (Table S1). In the Swiss Target Prediction database, 637 potential molecular targets (Table S2) of 97 compounds were collected (Figure 3A). In the DisGeNET database, 93 ALI-related genes were obtained (Table S3). Then, we screened 33 hub genes from upregulated genes, ALI-related genes, potential target genes of *E. bodinieri* Vaniot, and downregulated genes (Figure 3B). The GO network of hub genes was investigated by Metascape database, and Cytoscape software was utilized to construct a GO network that consisted of 163 nodes and 1967 edges (Figure 3C). Furthermore, based on potential target proteins and corresponding signaling pathways, a key genes-compounds network was constructed to further clarify the possible modes of action (Figure 3D). We analyzed the difference of *Ptgs2* and *Bcl2* between normal and ALI lung tissues at the transcription level by GSE1871, GSE17355, and GSE2411 datasets. The expression level of *Ptgs2* was significantly higher (*p* < 0.05, *p* < 0.01) in ALI than normal lung tissues (Figure 3E,F). Differently, *Bcl2* expression was markedly lower (*p* < 0.01) in ALI than in normal lung tissues (Figure 3G).

### 2.3. EBE Alleviated Oxidative Stress of Cells Model by NQO1

**Figure 4:**
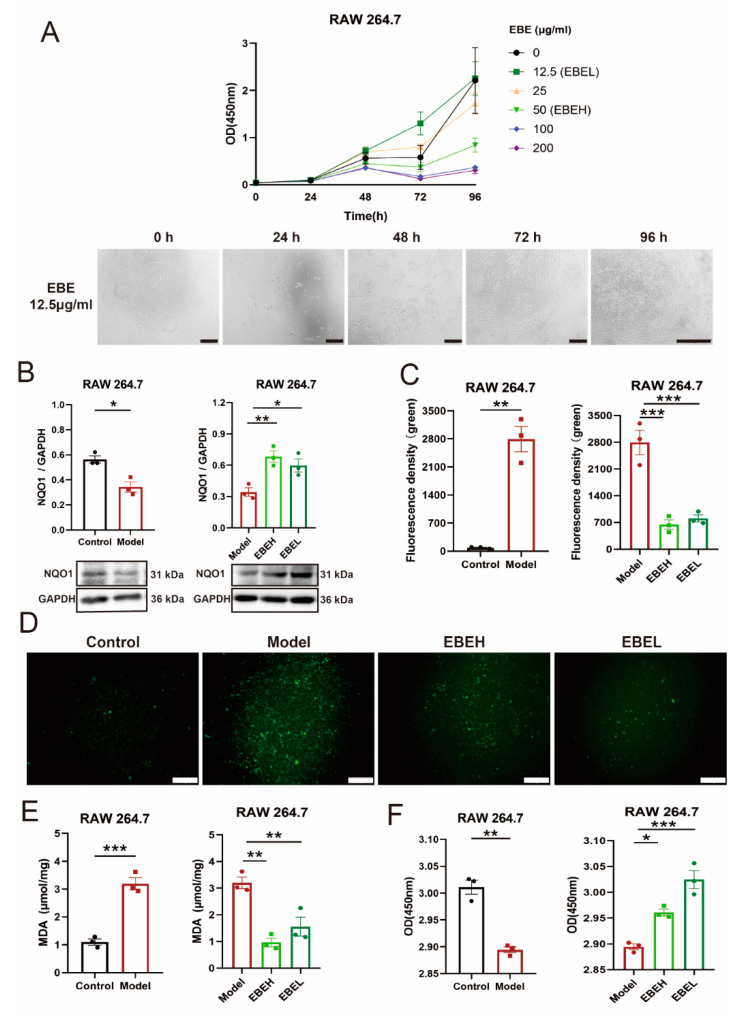
EBE alleviated oxidative stress of cells model by NQO1. (**A**) The effect of several concentrations of EBE on RAW 264.7 viability at different times. Scale bars of 0–72 h, 200 μm. Scale bars of 96 h, 400 μm. (**B**) NQO1 expression of cells for RAW 264.7 of vehicle treatment (Control), RAW 264.7 of LPS treatment (Model), model of 50 µg/mL EBE treatment (EBEH) and model of 12.5 µg/mL EBE treatment (EBEL), as measured by western blotting. (**C**) ROS content of cells for Control, Model, EBEH, and EBEL. (**D**) Representative fluorescence images of cells for Control, Model, EBEH, and EBEL. Green, ROS. Scale bar, 200 μm. (**E**,**F**) MDA level (**E**) and viability of cells (**F**) for Control, Model, EBEH, and EBEL. Unpaired *t*-test and ordinary one-way ANOVA were used on experimental data. Data are presented as mean ± SEM, *n* = 3. * *p* < 0.05, ** *p* < 0.01, *** *p* < 0.001.

To investigate the effect of EBE in vitro, gradient concentrations of EBE were determined at different times via Cell Counting Kit-8 (CCK-8). Then, 50 µg/mL EBE (EBE high concentration, EBEH) and 12.5 µg/mL EBE (EBE low concentration, EBEL) were used to explore effects on cells model (Figure 4A). We induced an inflammatory cells model by lipopolysaccharide (LPS) in RAW 264.7, NQO1 expression was shapely downregulated (*p* < 0.05) in cells model, EBEH and EBEL treatment both remarkably rescued NQO1 expression (*p* < 0.01, *p* < 0.05) (Figure 4B). Accordingly, reactive oxygen species (ROS), malondialdehyde (MDA) and cell viability in cells model were evaluated. In cells model, ROS content was marked accumulated (*p* < 0.01), comparatively, ROS content both significantly reduced (all *p* < 0.001) with EBEH and EBEL treatment (Figure 4C,D). Identically, the MDA level was remarkably swelled (*p* < 0.001), EBEH and EBEL treatment significantly diminished the MDA level (all *p* < 0.01) (Figure 4E). Consequently, cell viability was remarkably decreased (*p* < 0.01) in the cells model, EBEH and EBEL treatment all significantly increased (*p* < 0.05, *p* < 0.001) cell viability (Figure 4F).

### 2.4. EBE Inhibited Pyroptosis of Cells Model by BCL2

**Figure 5:**
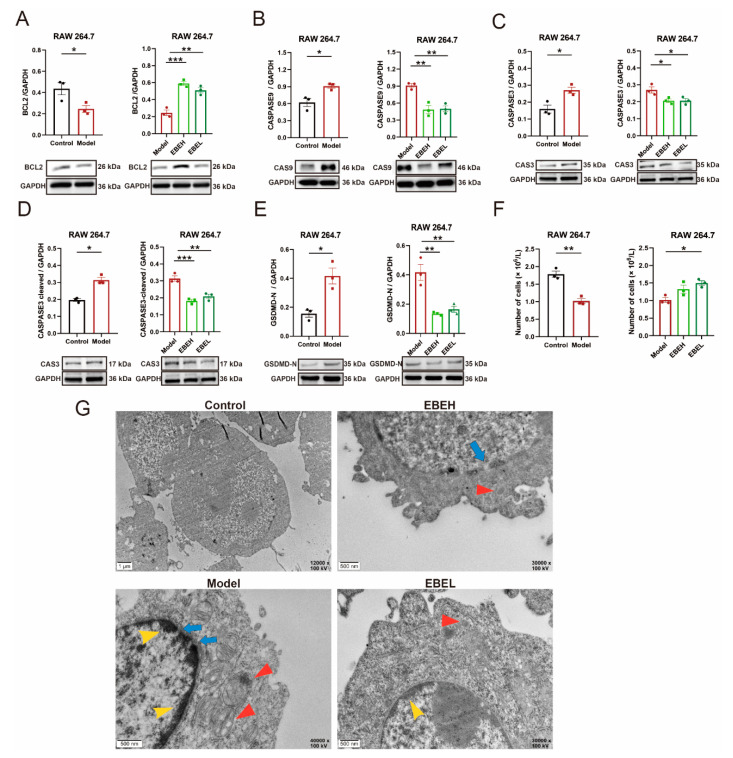
EBE inhibited pyroptosis of cells model by BCL2. (**A**–**F**) BCL2 (**A**), CASPASE9 (**B**), CASPASE3 (**C**), CASPASE3-cleaved (**D**), GSDMD-N (**E**), and numbers of living cells (**F**) for RAW 264.7 of vehicle treatment (Control), RAW 264.7 of LPS (Model), model of 50 µg/mL EBE treatment (EBEH) and model of 12.5 µg/mL EBE treatment (EBEL). (**G**) Transmission electron microscopy images of cells for Control, Model, EBEH and EBEL. Red arrows feature mitochondrial morphology, blue arrows feature membrane pores and yellow arrows feature heterochromatin marginalizing. (**A**–**E**) were measured by western blotting. Unpaired *t*-test and ordinary one-way ANOVA were used on experimental data. Data are presented as mean ± SEM, *n* = 3. * *p* < 0.05, ** *p* < 0.01, *** *p* < 0.001.

To examine whether EBE contributes to the inhibition of pyroptosis in ALI by BCL2, the protein expression levels of BCL2, CASPASE9, CASPASE3 and gasdermin D (GSDMD) were evaluated in vitro. BCL2 expression was downregulated (*p* < 0.05), EBEH and EBEL treatment significantly upregulated BCL2 levels (*p* < 0.001, *p* < 0.01) in cells model (Figure 5A). The CASPASE9 level was increased (*p* < 0.05), EBEH and EBEL treatment all decreased (all *p* < 0.01) it in cells model (Figure 5B). Accordingly, the CASPASE3 level was increased (*p* < 0.05), EBEH and EBEL treatment all decreased (all *p* < 0.05) in the cells model (Figure 5C). Further, we observed that the CASPASE3-cleaved level was increased (*p* < 0.05) by LPS, and were reversed (*p* < 0.001, *p* < 0.01) by EBEH and EBEL treatment in cells model (Figure 5D). Earlier studies demonstrated that the activation of the executioner CASPASE3 (CASPASE3-cleaved) promoted microglial pyroptosis by active GSDMD [17,18]. Next, we observed that the N-terminal fragments of GSDMD (GSDMD-N) level was significantly increased (*p* < 0.05), EBEH and EBEL treatment all decreased (all *p* < 0.01) the GSDMD-N level in the cells model (Figure 5E). As result, the number of living cells were remarkable decreased (*p* < 0.01) in the model, EBEL treatment significant restored (*p* < 0.05) numbers of living cells (Figure 5F).

In addition, transmission electron microscopy (TEM) results showed that mitochondrial swelling, mitochondrial cristae collapse, membrane pore formation and heterochromatin marginalizing in cells model, EBEH and EBEL treatment partially alleviated the abnormal changes of mitochondrial morphology, nuclear membrane and heterochromatin (Figure 5G). Collectively, these results showed that EBE suppressed pyroptosis of the cells model by upregulating the expression of BCL2.

### 2.5. EBE Attenuated Inflammation of Cells Model by PTGS2

**Figure 6:**
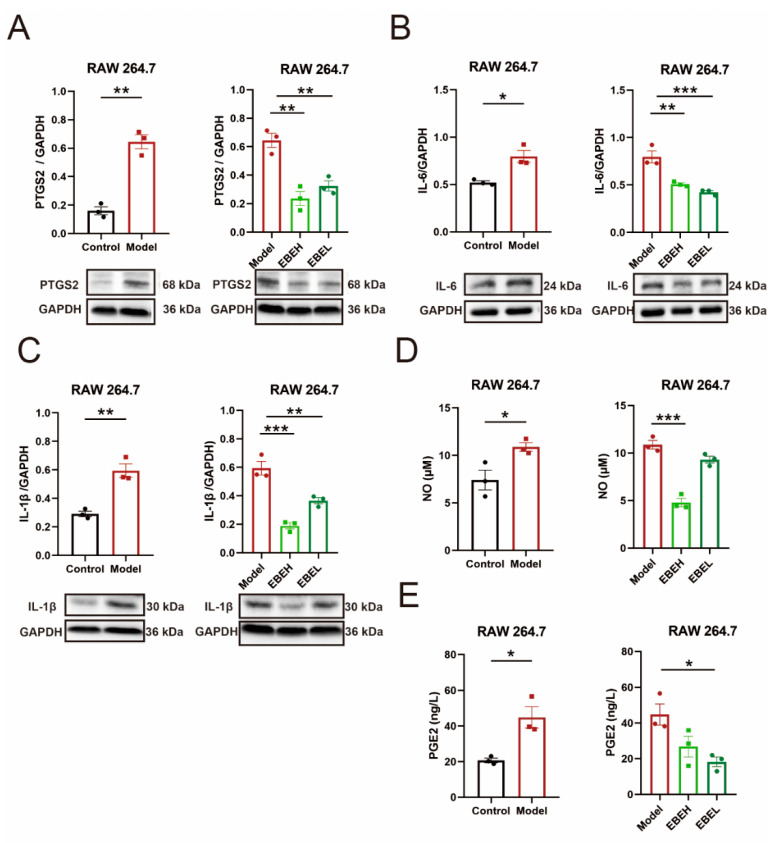
EBE attenuated inflammation of cells model by PTGS2. (**A**–**E**) PTGS2 (**A**), IL-6 (**B**), IL-1β (**C**), NO (**D**) and PGE2 (**E**) expression of cells for RAW 264.7 of vehicle treatment (Control), RAW 264.7 of LPS (Model), model of 50 µg/mL EBE treatment (EBEH), and model of 12.5 µg/mL EBE treatment (EBEL). (**A**–**C**) were measured by western blotting. Unpaired *t*-test and ordinary one-way ANOVA were used on experimental data. Data are presented as mean ± SEM, *n* = 3. * *p* < 0.05, ** *p* < 0.01, *** *p* < 0.001.

To determine whether EBE plays a basic role in the amelioration of inflammation in ALI by PTGS2, inflammatory factors and prostaglandin E2 (PGE2) were investigated in the cells model. PTGS2 expression was raised significantly (*p* < 0.01) in the cells model, but EBEH and EBEL treatment both reduced it (all, *p* < 0.01) (Figure 6A). Similarly, interleukin 6 (IL-6), interleukin 1 beta (IL-1β) and nitric oxide (NO) levels all were increased (*p* < 0.05, *p* < 0.01, *p* < 0.05) in the cells model; comparatively, EBEH and EBEL treatment significantly decreased IL-6 and IL-1β (*p* < 0.001, *p* < 0.01), but only EBEH treatment could markedly decrease NO (*p* < 0.001) (Figure 6B–D). Moreover, PGE2 level was increased (*p* < 0.05), EBEL treatment decreased (*p* < 0.05) it in the cells model (Figure 6E). These data indicated that EBE attenuated inflammation of the cells model by downregulating the expression of PTGS2.

## 3. Discussion

We retrieved 637 potential target genes regulated by *E. bodinieri* Vaniot in Swiss Target Prediction and 93 ALI-related genes in the DisGeNET database. These constituted the compilation of genes in ALI regulated by *E. bodinieri* Vaniot. GO and KEGG analysis were used to explore genes functions and signaling pathways. We analyzed different expression of *Ptgs2* and *Bcl2* between normal and ALI lung tissues at the transcription level by GSE1871, GSE17355, and GSE2411 datasets. *Nqo1* was a potential target gene regulated by terpenoids in EBE. As a key ROS scavenger, NQO1 contributes to alleviating oxidative stress [19,20,21]. Considering the above findings, we investigated *Bcl2*, *Ptgs2* and *Nqo1* as potential key genes in ALI regulated by *E. bodinieri* Vaniot. We verified these results by experiments in vitro.

Intensive oxidative stress is regarded as an important process to the pathogenesis of ALI [22]. Oxidative stress causes the damage and enzymolysis of cellular structural molecules, and finally induces apoptosis, necrosis, or other death modes [23]. We found that EBE treatment upregulated NQO1, decreased ROS and MDA content, and increased cell viability in the cells model. These findings indicated that EBE could alleviate oxidative stress of the cells model by upregulating NQO1 expression.

Pyroptosis, a programmed cell death pathway, is also known as inflammatory caspase-dependent cell death [24]. Pyroptosis can be triggered by noninfectious stimuli and microbes [25] and plays central regulatory roles in the progression of numerous diseases, such as ALI [26], Alzheimer’s disease [27], tumor [28], and inflammatory diseases [24]. We found that EBE contributed to the inhibition of pyroptosis and restoring numbers of living cells in the cells model. Mechanistically, the administration of EBE upregulated BCL2 expression, decreased CASPASE9, CASPASE3, CASPASE3-cleaved, and GSDMD-N levels in vitro. BCL2 promotes cellular survival by preserving the mitochondrial outer membrane, and inactivates the pyroptotic execution program [29,30]. We demonstrated that EBE treatment alleviated the abnormal changes of mitochondrial morphology, nuclear membrane and heterochromatin in cells model by TEM. We analyzed that *Bcl2* was the potential target gene regulated by EBE via bioinformatics methods. These experiments confirmed that EBE inhibited pyroptosis of cells models by BCL2.

The robust inflammatory response is another important pathological mechanism of ALI [31]. PTGS2 is induced to increase prostaglandins in response to inflammation and immune responses [32]. Previous research has suggested that the expression of PTGS2 was increased in ALI, and suppressing PTGS2 attenuated ALI induced by LPS [32,33,34]. Our results showed that administration of EBE downregulated PTGS2 expression, and decreased IL-6, IL-1β, NO and PGE2 levels in the cells model. These data verified that *Ptgs2* was a potential target gene regulated by EBE, and EBE attenuated inflammation of cells model by PTGS2. We also detected the alteration of TNFα, an important cytokine, but it was not significant in EBE treatment group (not exhibited), additionally, IFNγ is related to the innate and adaptive immune systems; maybe we will consider the effects of *E. bodinieri* Vaniot on IFNγ in animal models in our following work.

RAW 264.7 is a leukemic monocyte/macrophage cell line of mouse. Previous studies showed that RAW 264.7 was stimulated with LPS to establish a steadily inflammatory model [31]. In order to verify the bioinformatic results of mouse, RAW 264.7 was selected as an inflammatory cells model. However, lung endothelial barrier dysfunction is also an important pathological characteristic of ALI. To further explore the effects of *E. bodinieri* Vaniot on ALI lung tissue, we will continue working on alveolar epithelial cell lines and animal models.

Failure to resolve the ALI worsening will increase the poor prognosis and mortality of patients [35]. Although a large number of ALI research has been conducted, no effective drug treatments that significantly decrease the mortality have been reported [36]. We aim to explore natural products from *E. bodinieri* Vaniot that could ameliorate ALI, and serve as a functional food for adjuvant therapy or as a therapeutic agent.

## 4. Materials and Methods

### 4.1. ALI Gene Expression Data Acquisition and Processing

The mRNA expression data of ALI and normal lung tissue were obtained from the GEO database (http://www.ncbi.nlm.nih.gov/geo/). We collected 9 mouse ALI samples and 3 matched control samples in GSE17355 microarray data [37], 6 mouse ALI samples and 6 control samples in GSE2411 [38], and 3 mouse lung samples treated with LPS and 3 corresponded controls in GSE1871 [39]. In order to obtain DEGs of ALI, we used the Limma R package [40] to analyze GSE17355, GSE2411 and GSE1871 datasets based on a criterion of *p* < 0.05 after adjustment by the false discovery rate (FDR). The clusterProfiler, org.Mm.eg.db, enrichplot, and ggplot2 R packages were used to determine the GO biological processes analysis of overlapping DEGs. To uncover the potential biological functions and interactive networks of overlapping DEGs, KEGG pathway (https://www.genome.jp/kegg/) enrichment analysis was performed using the clusterProfiler R package.

### 4.2. Exploration and Identification of Key Genes

Compounds of *E. bodinieri* Vaniot were retrieved in CAS SciFinder database (https://accounts.cas.org/products/) [41] and PubChem database (https://pubchem.ncbi.nlm.nih.gov/) [42]. The associated target genes of *E. bodinieri* Vaniot compounds were predicted and obtained from Swiss Target Prediction database (http://www.swisstargetprediction.ch/) [43]. ALI-related genes were obtained in DisGeNET database (https://www.disgenet.org/) [44]. We screened hub genes from upregulated genes, ALI-related genes, potential target genes of *E. bodinieri* Vaniot and downregulated genes by VennDiagram R package. GO network of hub genes was investigated by Metascape database (https://metascape.org/) [45], and Cytoscape software (Version: 3.8.0) was utilized to construct a GO network. The genes-compounds network was constructed with Cytoscape software 3.8.0 based on potential target proteins and corresponding signaling pathways.

### 4.3. Cell Experiments 

RAW264.7 cells (TIB-71, ATCC, Rockefeller, MA, USA), mouse mononuclear macrophage leukemia cells, were cultured in DMEM/high glucose (06-1055-57-1A, Biological Industries, Kibbutz Beit-Haemek, Israel) supplemented with 10% fetal bovine serum (04-001-1A, Biological Industries, Kibbutz Beit-Haemek, Israel), 100 µg/mL streptomycin, and 100 U/mL penicillin (P1400, Solarbio, Beijing, China) at 37 °C in 5% CO_2_ humidified atmosphere. The cells were passaged every 1–2 days upon approaching confluency.

RAW264.7 cells were seeded at a density of 2.65 × 10^5^ cells/well in 6-well cell culture plates, and were stimulated with LPS (1 μg/mL) for 24 h to establish a steadily inflammatory model [31]. According to the instructions, LPS (L8880, Solarbio, Beijing, China) 1 ng is equivalent to 0.5 EU (tachypleus amebocyte lysate method) and 10 EU (chromogenic method). The dose of LPS is 1 µg/mL, which is equivalent to 500 EU/mL (tachypleus amebocyte lysate method) and 10,000 EU/mL (chromogenic method). Our previous studies showed that nine principal compounds were identified from EBE by UHPLC-ESI-HRMS/MS analysis. According to the structural characteristics, the identified compounds included 6 flavanone glycosides (3, 4, 5, 6, 7 and 9) and 3 phenolic acids (1, 2, and 8) (Table 1). The cells were divided into four groups: LPS was added to the cells after incubation with 50 μg/mL EBE (EBEH) for 24 h; LPS was added to the cells after incubation with 12.5 μg/mL EBE (EBEL) for 24 h; LPS was added to the cells without EBE (Model); nothing was added to the cells without EBE (Control). The viability of RAW 264.7 cells was performed by CCK-8 assay (CK04, DOJINDO, Kumamoto, Japan).

### 4.4. Detection of Inflammatory Cytokines and Oxidative Stress Related Factors 

The levels of NO (S0021S, Beyotime, Shanghai, China) and PGE2 (H099-1, Nanjing Jiancheng, Nanjing, China) were assessed by the appropriate kits according to the manufacturer’s instructions. The contents of MDA (S0131S, Beyotime, Shanghai, China) and ROS (S0033S, Beyotime, Shanghai, China) were determined according to the instructions of the kits. Data were read on a microplate reader (Biotek, Santa Clara, CA, USA). ROS fluorescent probe were photographed under a fluorescence microscope (Olympus, Tokyo, Japan) and analyzed using Image-Pro Plus 6.0 software.

### 4.5. Transmission Electron Microscopy 

The cells were pelleted by centrifugation at 3000 rpm and then fixed in 2.5% glutaraldehyde cold fixing solution for 2 h. The cell mass was sliced to small pieces and pre-fixed in 2.5% glutaraldehyde PBS solution on ice for 2 h. The primarily fixed samples were further sliced to a volume no larger than 2 mm, rinsed with PBS for three times and then post-fixed in 1% osmium tetroxide for 1 h. After dehydration in an ascending gradient ethanol solution, the samples were finally embedded in epoxy resin. Ultrathin sections were stained with uranyl acetate and lead citrate and examined with TEM (JEOL, Tokyo, Japan).

### 4.6. Western Blotting

RAW264.7 cells were lysed on ice using the RIPA lysis buffer (R0010, Solarbio, Beijing, China). The protein concentration in the lysis buffer was determined by BCA protein quantification kit (P0010, Beyotime, Shanghai, China). Equal amounts of proteins were separated by SDS-PAGE (10–12%) and transferred to PVDF membrane (IPVH00010, Millipore, Saint Louis, MO, USA). The membranes were incubated with primary antibody: NQO1 (67240-1-lg, Proteintech, Wuhan, China), BCL2 (66799-1-lg, Proteintech, Wuhan, China), CASPASE9 (ab202068, Abcam, Boston, MA, USA), CASPASE3 (19677-1-AP, Proteintech, Wuhan, China), GSDMD (20770-1-AP, Proteintech, Wuhan, China), PTGS2 (12375-1-AP, Proteintech, Wuhan, China), IL-6 (ab7737, Abcam, Boston, MA, USA), IL-1β (ab9722, Abcam, Boston, MA, USA), GAPDH (60004-1-lg, Proteintech, Wuhan, China). Anti-mouse lgG (SA00001-1, Proteintech, Wuhan, China) and anti-rabbit lgG (SA00001-2, Proteintech, Wuhan, China) were the secondary antibodies.

### 4.7. Statistical Analysis

All results are presented as the mean ± SEM. A two-tailed unpaired Student’s *t*-test (for two group comparison) and a one-way ANOVA followed by Turkey’s post-hoc test (for multi-group comparison) were performed using GraphPad Prism v.8.0.1; *p* value < 0.05 was considered statistically significant. All experiments were repeated independently at least three times with similar results.

## 5. Conclusions

In conclusion, we used bioinformatics methods to explore and analyze potential target genes in ALI regulated by *E. bodinieri* Vaniot. We confirmed the effects of *E. bodinieri* Vaniot on cells model of ALI by determining oxidative stress, pyroptosis and inflammation in vitro. This study provides a potential natural source of ameliorating ALI for functional food and pharmaceutical applications.

## Figures and Tables

**Table 1:** Main chemical components of EBE. The information of the EBE compounds identified by the UHPLC-ESI-HRMS/MS.

No.	Compounds	Molecular	Classification
1	Quinic acid	C_7_H_12_O_6_	Phenolic acid
2	Chlorogenic acid	C_16_H_18_O_9_	Phenolic acid
3	Apigenin 6-C-glucoside 8-C-arabinoside	C_26_H_28_O_14_	Flavanone glycosides
4	Luteolin-7-O-rutinoside	C_27_H_30_O_15_	Flavanone glycosides
5	Eriodictyol-7-O-glucoside	C_21_H_22_O_11_	Flavanone glycosides
6	Luteolin-7-O-glucoside	C_21_H_20_O_11_	Flavanone glycosides
7	Apigenin-7-O-glucoside	C_21_H_20_O_10_	Flavanone glycosides
8	Rosmarinic acid	C_18_H_16_O_8_	Phenolic acid
9	(2R)Eriodictyol 7-O-(6″-3,4-dihydroxycinnamoyl)-β-D-glucopyranoside	C_30_H_28_O_14_	Flavanone glycosides

## Data Availability

The data supporting this study’s findings are available upon request.

## Supplementary Materials

The following supporting information can be downloaded at: https://www.mdpi.com/article/10.3390/ijms232415651/s1.
