# Supplementary material for: Elsholtzia bodinieri Vaniot Ameliorated Acute Lung Injury by NQO1, BCL2 and PTGS2 In Silico and In Vitro Analyses
Source: Int J Mol Sci. 2022 Dec 9;23(24):15651. doi: 10.3390/ijms232415651 (PMC9779453; doi:10.3390/ijms232415651)
Supplement: Supplementary file 1 [file ijms-23-15651-s001.zip › ijms-1977897-supplementary.pdf]

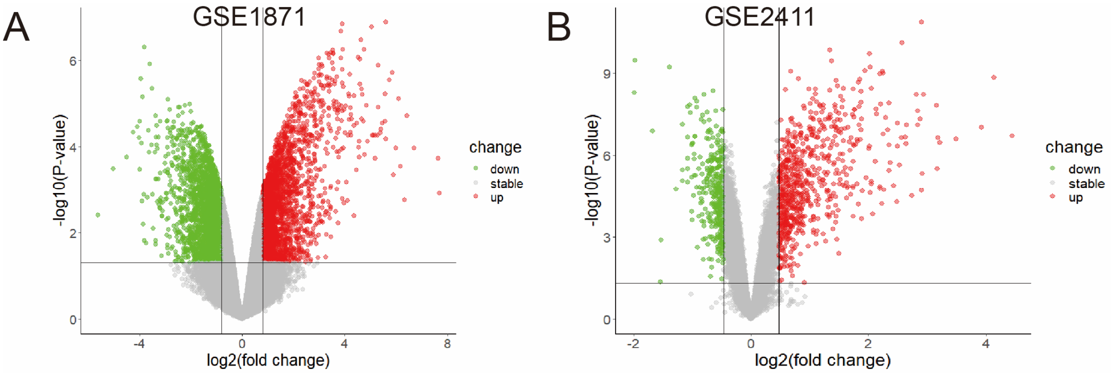

Figure S1. Transcriptomic differentially expressed genes of ALI (related to Fig. 1). (A) Volcano plot showing DEGs between ALI and normal lung tissue in GSE1871 dataset. (B) Volcano plot showing DEGs between ALI and normal lung tissue in GSE2411 dataset. There red dots indicate upregulated genes, green dots indicate downregulated genes, and gray dots indicate stable genes (corrected P values <0.05).

Table S1. Compounds of *E. bodinieri* Vaniot. 97 compounds of *E. bodinieri* Vaniot were retrieved in CAS SciFinder database and PubChem database.

Compound	Molecular Formula	Molecular Weight	CAS ID	Reference
Cynaroside	$C_{21}H_{20}O_{11}$	448.4	5373/11/5	DOI:10.1177/1934578x1801301122
5Z-Dodecenyl Acetate	$C_{14}H_{26}0_2$	226.35	16676-96-3	DOI:CNKI:SUN:SPYK.0.2020-04-035(Chinese)
P-Cymen-8-Ol	$C_{10}H_{14}O$	150.22	1197-01-9	DOI:10.1080/14786419.2017.1395436
Vinyl Amyl Carbinol	$C_8H_{16}O$	128.21	3391-86-4	DOI:10.3969/j.issn.1007-855x.2010.01.018(Chinese)
(+)-Carotol	$C_{15}H_{26}O$	222.37	465-28-1	DOI:CNKI:SUN:SPYK.0.2020-04-035(Chinese)
(+/-)-Eriodictyol	$C_{15}H_{12}O_6$	288.25	552-58-9	DOI:CNKI:CDMD:1.1015.528521(Chinese)
(1R,2R,4R)-Trihydroxy-p-menthane	$C_{10}H_{20}O_3$	188.26	2514-94-5	DOI:10.3969/j.issn.1007-855x.2013.05.013(Chinese)
1-Carvone	$C_{10}H_{14}O$	150.22	2244-16-8	DOI:10.3969/j.issn.1007-855x.2010.01.018(Chinese)
1,8-Cineole	$C_{10}H_{18}O$	154.25	470-82-6	DOI:CNKI:SUN:SPYK.0.2020-04-035(Chinese)
2- Dodecenal	$C_{12}H_{22}O$	182.3	4826-62-4	DOI:CNKI:SUN:SPYK.0.2020-04-035(Chinese)
2-Pinene	$C_{10}H_{16}$	136.23	80-56-8	DOI:10.3969/j.issn.1007-855x.2010.01.018(Chinese)
2α,19α-Dihydroxyursolic acid	$C_{30}H_{48}O_5$	488.7	13850-16-3	DOI:10.1002/hlca.200590006
3-cyclohexene-1-methanol	$C_{10}H_{18}O$	154.25	98-55-5	DOI:10.3969/j.issn.1007-855x.2010.01.018(Chinese)
3,4-Dihydroxy-5-Methoxybenzaldehyde	$C_8H_8O_4$	168.15	3934-87-0	DOI:10.3969/j.issn.1007-855x.2013.05.013(Chinese)
3,7-dimethylocta-2,6-dienyl propanoate	$C_{13}H_{22}O_2$	210.31	152481-48-6	DOI:10.3969/j.issn.1007-855x.2010.01.018(Chinese)
4-Isopropylbenzaldehyde	$C_{10}H_{12}O$	148.2	122-03-2	DOI:10.3969/j.issn.1007-855x.2010.01.018(Chinese)
4-Isopropylbenzyl alcohol	$C_{10}H_{14}O$	150.22	536-60-7	DOI:10.1007/s00217-021-03829-4
Acacetin	$C_{16}H_{12}O_5$	284.26	480-44-4	DOI:10.13467/j.cnki.jbuns.2006.03.009(Chinese)
alpha-Caryophyllene	$C_{15}H_{24}$	204.35	6753-98-6	DOI:10.3969/j.issn.1007-855x.2010.01.018(Chinese)
alpha-Humulene	$C_{15}H_{24}$	204.35	6753-98-6	DOI:10.3969/j.issn.1007-855x.2010.01.018(Chinese)
alpha-Muurolene	$C_{15}H_{24}$	204.35	10208-80-7	DOI:CNKI:SUN:SPYK.0.2020-04-035(Chinese)
ALPHA-PINENE	$C_{10}H_{16}$	136.23	80-56-8	DOI:10.3969/j.issn.1007-855x.2010.01.018(Chinese)
Amentoflavone	$C_{30}H_{18}O_{10}$	538.5	1617-53-4	DOI:10.13863/j.issn1001-4454.2005.02.009(Chinese)
Apigenin	$C_{15}H_{10}O_5$	270.24	520-36-5	DOI:10.1248/cpb.56.592
Arachidic acid	$C_{20}H_{40}O_2$	312.5	506-30-9	DOI:10.3321/j.issn:1673-1689.2008.02.013(Chinese)
beta-Caryophyllene	$C_{15}H_{24}$	204.35	87-44-5	DOI:10.3969/j.issn.1007-855x.2010.01.018(Chinese)
BETA-TERPINEOL	$C_{10}H_{18}O$	154.25	138-87-4	DOI:CNKI:SUN:CYKX.0.2007-08-008(Chinese)
Bicyclo[3,1,1]heptan-3-ol	$C_{10}H_{16}O$	152.23	1674-08-4	DOI:10.3969/j.issn.1007-855x.2010.01.018(Chinese)
Bodinioside A	$C_{36}H_{58}O_{12}$	682.8	851120-60-0	DOI:10.1002/hlca.200590006
Bodinioside B	$C_{36}H_{58}O_{11}$	666.8	851120-62-2	DOI:10.1002/hlca.200590006
Bornyl acetate	$C_{12}H_{20}O_2$	196.29	5655-61-8	DOI:10.3969/j.issn.1007-855x.2010.01.018(Chinese)
caffeic acid	$C_9H_8O_4$	180.16	331-39-5	DOI:10.3969/j.issn.1007-855x.2013.05.013(Chinese)
Carotol	$C_{15}H_{26}O$	222.37	465-28-1	DOI:CNKI:SUN:SPYK.0.2020-04-035(Chinese)
Carvone Hydrate	$C_{10}H_{18}O_3$	186.25	60593-11-5	DOI:CNKI:CDMD:1.1015.528521(Chinese)
Caryophyllene	$C_{15}H_{24}$	204.35	87-44-5	DOI:10.3969/j.issn.1007-855x.2010.01.018(Chinese)
Caryophyllene oxide	$C_{15}H_{24}O$	220.35	1139-30-6	DOI:10.3969/j.issn.1007-855x.2010.01.018(Chinese)
CAS 1038743-23-5	$C_{31}H_{30}O_{14}$	626.6	1038743-23-5	DOI:10.1248/cpb.56.592
Chrysin	$C_{15}H_{10}O_4$	254.24	480-40-0	DOI:10.1248/cpb.56.592

Cineole	$C_{10}H_{18}O$	154.25	470-82-6	DOI:CNKI:SUN:SPYK.0.2020-04-035(Chinese)
Coleonolic acid	$C_{30}H_{46}O_4$	470.7	128397-09-1	DOI:10.1002/hlca.200590006
Cosmosiine	$C_{21}H_{20}O_{10}$	432.4	578-74-5	DOI:10.1080/14786419.2016.1164698
Cyclohexane	C_6H_{12}	84.16	110-82-7	DOI:10.3969/j.issn.1007-855x.2010.01.018(Chinese)
D-Myrtenal	$C_{10}H_{14}O$	150.22	564-94-3	DOI:10.3969/j.issn.1007-855x.2010.01.018(Chinese)
Daucosterol	$C_{35}H_{60}O_6$	576.8	474-58-8	DOI:10.13863/j.issn1001-4454.2005.02.009(Chinese)
Decanoic acid	$C_{10}H_{20}O_2$	172.26	334-48-5	DOI:CNKI:SUN:SPYK.0.2020-04-035(Chinese)
Decyl acetate	$C_{12}H_{24}O_2$	200.32	112-17-4	DOI:CNKI:SUN:SPYK.0.2020-04-035(Chinese)
Dimethyl 2-methylene bicyclo-[2.2.1] heptan-3-one	$C_{10}H_{14}O$	150.22	19890-00-7	DOI:10.3969/j.issn.1007-855x.2010.01.018(Chinese)
DL-Catechin	$C_{15}H_{14}O_6$	290.27	154-23-4	DOI:10.1080/10408690390826464
Durenol	$C_{10}H_{14}O$	150.22	527-35-5	DOI:10.7506/spkx1002-6630-201402045(Chinese)
Fenchol	$C_{10}H_{18}O$	154.25	1632-73-1	DOI:10.3969/j.issn.1007-855x.2010.01.018(Chinese)
Gallic acid	$C_7H_6O_5$	170.12	149-91-7	DOI:10.3321/j.issn:0253-2670.2007.03.003(Chinese)
Gallocatechol	$C_{15}H_{14}O_7$	306.27	970-73-0	DOI:10.13467/j.cnki.jbuns.2006.03.009(Chinese)
Geranyl acetate	$C_{12}H_{20}O_2$	196.29	105-87-3	DOI:10.3969/j.issn.1007-855x.2010.01.018(Chinese)
Globulol	$C_{15}H_{26}O$	222.37	489-41-8	DOI:10.3969/j.issn.1007-855x.2010.01.018(Chinese)
Icariside E4	$C_{26}H_{34}O_{10}$	506.5	126253-42-7	DOI:CNKI:CDMD:1.1015.528521(Chinese)
Kaempferol	$C_{15}H_{10}O_6$	286.24	520-18-3	DOI:10.3321/j.issn:0253-2670.2007.03.003(Chinese)
L-Alpha-Cadinol	$C_{15}H_{26}O$	222.37	481-34-5	DOI:10.11842/wst.20190307002(Chinese)
L-Carveol	$C_{10}H_{16}O$	152.23	99-48-9	DOI:10.3969/j.issn.1007-855x.2010.01.018(Chinese)
Ledol	$C_{15}H_{26}O$	222.37	577-27-5	DOI:10.3969/j.issn.1007-855x.2010.01.018(Chinese)
Limonene	$C_{10}H_{16}$	136.23	138-86-3	DOI:10.13863/j.issn1001-4454.2020.04.020(Chinese)
Linalool	$C_{10}H_{18}O$	154.25	78-70-6	DOI:CNKI:SUN:SPYK.0.2020-04-035(Chinese)
Luteolin	$C_{15}H_{10}O_6$	286.24	491-70-3	DOI:10.1080/14786419.2016.1164698
Luteolin 7-(6''-ferulylglucoside)	$C_{31}H_{28}O_{14}$	624.5	98767-38-5	DOI:10.1248/cpb.56.592
m-alpha-Terpineol, acetate	$C_{12}H_{20}O_2$	196.29	80-26-2	DOI:CNKI:SUN:SPYK.0.2020-04-035(Chinese)
Maslinic acid	$C_{30}H_{48}O_4$	472.7	4373-41-5	DOI:10.1080/14786419.2016.1164698
Methyl caffeate	$C_{10}H_{10}O_4$	194.18	3843-74-1	DOI:CNKI:CDMD:1.1015.528521(Chinese)
Methyl geranate	$C_{11}H18O_2$	182.26	1189-09-9	DOI:CNKI:SUN:SPYK.0.2020-04-035(Chinese)
Miscanthoside	$C_{21}H_{22}O_{11}$	450.4	38965-51-4	DOI:10.7523/j.issn.2095-6134.2008.4.020(Chinese)
Myrtenol	$C_{10}H_{16}O$	152.23	515-00-4	DOI:10.13863/j.issn1001-4454.2020.04.020(Chinese)
Naphthalene	$C_{10}H_8$	128.169	91-20-3	DOI:10.13989/j.cnki.0517-6611.2010.10.155(Chinese)
Nepetoidin B	$C_{17}H_{14}O_6$	314.29	55486-06-1	DOI: 10.1080/14786419.2016.1164698
Nerol	$C_{10}H_{18}O$	154.25	106-25-2	DOI:CNKI:SUN:SPYK.0.2020-04-035(Chinese)
Neryl Acetate	$C_{12}H_{20}O_2$	196.29	141-12-8	DOI:CNKI:SUN:SPYK.0.2020-04-035(Chinese)
O-CYMENE	$C_{10}H_{14}$	134.22	527-84-4	DOI:10.13989/j.cnki.0517-6611.2009.18.094(Chinese)
Oblonganoside I	$C_{41}H_{66}O_{14}$	783		DOI: 10.1080/14786419.2018.1477144
Oleanolic acid	$C_{30}H_{48}O_3$	456.7	508-02-1	DOI: 10.1080/14786419.2016.1164698
Palmitic Acid	$C_{16}H_{32}O_2$	256.42	57-10-3	DOI:CNKI:SUN:SPYK.0.2020-04-035(Chinese)
Perillyl acetate	$C_{12}H_{18}O_2$	194.27	15111-96-3	DOI:CNKI:SUN:SPYK.0.2020-04-035(Chinese)
Perillyl alcohol	$C_{10}H_{16}O$	152.23	536-59-4	DOI:CNKI:SUN:SPYK.0.2020-04-035(Chinese)
PHYTONE	$C_{18}H_{36}O$	268.5	502-69-2	DOI:CNKI:SUN:SPYK.0.2020-04-035(Chinese)
Protocatechualdehyde	$C_7H_6O_3$	138.12	139-85-5	DOI:10.3969/j.issn.1007-855x.2013.05.013(Chinese)

PubChem CID 21637755	$C_{42}H_{68}O_{16}$	829		DOI: 10.1080/14786419.2018.1477144
Quercetin	$C_{15}H_{10}O_7$	302.23	117-39-5	DOI:10.13467/j.cnki.jbuns.2006.03.009(Chinese)
Rosmarinic acid	$C_{18}H_{16}O_8$	360.3	20283-92-5	DOI:10.3969/j.issn.1007-855x.2013.05.013(Chinese)
Rosmarinic acid ethyl ester	$C_{20}H_{20}O_8$	388.4	174591-47-0	DOI:10.3969/j.issn.1007-855x.2013.05.013(Chinese)
Rosmarinic acid methyl ester	$C_{19}H_{18}O_8$	374.3	99353-00-1	DOI:10.3969/j.issn.1007-855x.2013.05.013(Chinese)
Rotungenoside	$C_{36}H_{58}O_{10}$	650	121387-38-0	DOI:10.1080/14786419.2018.1477144
Spathulenol	$C_{15}H_{24}O$	220.35	6750-60-3	DOI:10.3969/j.issn.1007-855x.2010.01.018(Chinese)
Terpinen-4-ol	$C_{10}H_{18}O$	154.25	562-74-3	DOI:10.3969/j.issn.1007-855x.2010.01.018(Chinese)
Terpineol	$C_{10}H_{18}O$	154.25	98-55-5	DOI:CNKI:SUN:SPYK.0.2020-04-035(Chinese)
Terpinyl Acetate	$C_{12}H_{20}O_2$	196.29	80-26-2	DOI:CNKI:SUN:SPYK.0.2020-04-035(Chinese)
Thymol	$C_{10}H_{14}O$	150.22	89-83-8	DOI:CNKI:SUN:CYKX.0.2007-08-008(Chinese)
Torreyol	$C_{15}H_{26}O$	222.37	19435-97-3	DOI:CNKI:SUN:SPYK.0.2020-04-035(Chinese)
Tricin	$C_{17}H_{14}O_7$	330.29	520-32-1	DOI:10.13467/j.cnki.jbuns.2006.03.009(Chinese)
Vanillin	$C_8H_8O_3$	152.15	121-33-5	DOI:10.3321/j.issn:0253-2670.2007.03.003(Chinese)
VERBENOL	$C_{10}H_{16}O$	152.23	473-67-6	DOI:10.3969/j.issn.1007-855x.2010.01.018(Chinese)
β-sitosterol	$C_{29}H_{50}O$	414.7	83-46-5	DOI:10.13863/j.issn1001-4454.2005.02.009(Chinese)

Table S2. Potential molecular targets of compounds in *E. bodinieri* Vaniot. In Swiss Target Prediction database, 637 potential molecular targets of 97 compounds were collected.

Compound	Target Gene	Uniprot ID	ChEMBL ID	Probability*	Known actives (3D/2D)
Methyl caffeate	ABAT	P80404	CHEMBL2044	0.031227	3 / 0
Cosmosiine	ABCB1	P08183	CHEMBL4302	0.118883	0 / 44
CAS 1038743-23-5	ABCC1	P33527	CHEMBL3004	0.055648	6 / 0
Geranyl acetate	ABCC9	O60706	CHEMBL1971	0.031227	2 / 0
CAS 1038743-23-5	ABCG2	Q9UNQ0	CHEMBL5393	0.055648	0 / 11
Geranyl acetate	ABHD6	Q9BV23	CHEMBL2189127	0.031227	5 / 0
Gallocatechol	ABL1	P00519	CHEMBL1862	0.101614	142 / 0
Phytone	ACACA	Q13085	CHEMBL3351	0.112042	75 / 0
Phytone	ACACB	O00763	CHEMBL4829	0.112042	262 / 0
Coleonolic acid	ACE	P12821	CHEMBL1808	0.120226	223 / 0
3,4-Dihydroxy-5-methoxybenzaldehyde	ACHE	P22303	CHEMBL220	0.125076	5 / 0
Carvone Hydrate	ACP1	P24666	CHEMBL4903	0.125076	0 / 1
Vinyl Amyl Carbinol	ACPP	P15309	CHEMBL2633	0.023833	6 / 0
Rosmarinic acid ethyl ester	ADAM17	P78536	CHEMBL3706	0.115737	386 / 0
2- Dodecenal	ADH1A	P07327	CHEMBL1970	0.071787	10 / 0
D-Myrtenal	ADH1B	P00325	CHEMBL3284	0.043919	7 / 0
2- Dodecenal	ADH1C	P00326	CHEMBL3285	0.071787	3 / 0
4-Isopropylbenzaldehyde	ADH4	P08319	CHEMBL2990	0.053518	1 / 0
Rosmarinic acid	ADK	P55263	CHEMBL3589	0.10934	45 / 0
Cosmosiine	ADORA1	P30542	CHEMBL226	0.215357	45 / 1
CAS 1038743-23-5	ADORA2A	P29274	CHEMBL251	0.055648	33 / 1
Daucosterol	ADORA2B	P29275	CHEMBL255	0.074392	6 / 0
Carvone Hydrate	ADORA3	P0DMS8	CHEMBL256	0.125076	0 / 3
3,7-Dimethylocta-2,6-dienyl propanoate	ADRA1A	P35348	CHEMBL229	0.042894	1 / 0
Geranyl acetate	ADRA1D	P25100	CHEMBL223	0.031227	20 / 0
Decyl acetate	ADRA2A	P08913	CHEMBL1867	0.031227	21 / 0
Terpinyl Acetate	ADRA2B	P18089	CHEMBL1942	0.031227	19 / 0
Cosmosiine	ADRA2C	P18825	CHEMBL1916	0.118883	19 / 0
Coleonolic acid	AGTR1	P30556	CHEMBL227	0.120226	123 / 0
Maslinic acid	AGTR2	P50052	CHEMBL4607	0.120226	17 / 0
Apigenin	AHR	P35869	CHEMBL3201	0.272563	1 / 1
Kaempferol	AKR1A1	P14550	CHEMBL2246	0.402643	1 / 1
Caffeic acid	AKR1B1	P15121	CHEMBL1900	0.150924	23 / 30
Carvone Hydrate	AKR1B10	O60218	CHEMBL5983	0.166646	0 / 6
5Z-Dodecenyl Acetate	AKR1C1	Q04828	CHEMBL5905	0.060425	0 / 1
5Z-Dodecenyl Acetate	AKR1C2	P52895	CHEMBL5847	0.060425	0 / 1
Rosmarinic acid	AKR1C3	P42330	CHEMBL4681	0.10934	2 / 20
Caffeic acid	AKR1C4	P17516	CHEMBL4999	0.071787	0 / 1
Apigenin	AKT1	P31749	CHEMBL4282	0.108771	0 / 16
Thymol	ALB	P02768	CHEMBL3253	0.043919	0 / 5

Geranyl acetate	ALDH1A1	P00352	CHEMBL3577	0.031227	10 / 0
Cosmosiine	ALDH2	P05091	CHEMBL1935	0.118883	10 / 0
Geranyl acetate	ALDH3A1	P30838	CHEMBL3578	0.031227	5 / 0
Methyl caffeate	ALDH5A1	P51649	CHEMBL1911	0.031227	1 / 0
Gallic acid	ALK	Q9UM73	CHEMBL4247	0.125076	0 / 3
Apigenin	ALOX12	P18054	CHEMBL3687	0.47744	1 / 0
Rosmarinic acid ethyl ester	ALOX15	P16050	CHEMBL2903	0.115737	17 / 0
Carvone Hydrate	ALOX5	P09917	CHEMBL215	0.116739	13 / 20
Rosmarinic acid ethyl ester	ALOX5AP	P20292	CHEMBL4550	0.115737	164 / 0
Vanillin	ALPG	P10696	CHEMBL3402	0.043919	2 / 0
Phytone	ALPL	P05186	CHEMBL5979	0.112042	3 / 0
2α,19α-Dihydroxyursolic acid	AMPD2	Q01433	CHEMBL2997	0.110612	12 / 0
Rosmarinic acid	AMPD3	Q01432	CHEMBL2912	0.10934	18 / 0
Apigenin	AMY1A	P04745	CHEMBL2478	0.329868	1 / 1
Rosmarinic acid methyl ester	ANPEP	P15144	CHEMBL1907	0.113286	24 / 0
Carvone Hydrate	AOC3	Q16853	CHEMBL3437	0.116739	31 / 0
Chrysin	APEX1	P27695	CHEMBL5619	0.119895	0 / 1
Caffeic acid	APP	P05067	CHEMBL2487	0.071787	0 / 44
Carvone Hydrate	AR	P10275	CHEMBL1871	0.158398	0 / 50
Apigenin	ARG1	P05089	CHEMBL1075097	0.370888	2 / 2
Decyl acetate	ASAH1	Q13510	CHEMBL5463	0.031227	29 / 0
Carvone Hydrate	ATP12A	P54707	CHEMBL2933	0.133391	0 / 9
Rosmarinic acid ethyl ester	AURKA	O14965	CHEMBL4722	0.115737	27 / 0
Rosmarinic acid ethyl ester	AURKB	Q96GD4	CHEMBL2185	0.115737	141 / 0
Apigenin	AVPR2	P30518	CHEMBL1790	0.108771	3 / 1
Rotungenoside	AXL	P30530	CHEMBL4895	0.035898	17 / 0
Carvone Hydrate	BACE1	P56817	CHEMBL4822	0.141787	0 / 1
Decanoic acid	BCAT2	O15382	CHEMBL3616354	0.125076	2 / 0
Carvone Hydrate	BCHE	P06276	CHEMBL1914	0.133391	0 / 2
5Z-Dodecenyl Acetate	BCL2	P10415	CHEMBL4860	0.060425	31 / 0
PubChem CID 21637755	BCL2L1	Q07817	CHEMBL4625	0.079146	1 / 3
Rosmarinic acid ethyl ester	BMP1	P13497	CHEMBL3898	0.115737	236 / 0
Rosmarinic acid ethyl ester	BRAF	P15056	CHEMBL5145	0.115737	131 / 0
Carvone Hydrate	BRD2	P25440	CHEMBL1293289	0.125076	9 / 0
Carvone Hydrate	BRD4	O60885	CHEMBL1163125	0.125076	40 / 0
Geranyl acetate	C1R	P00736	CHEMBL4611	0.031227	22 / 0
Gallocatechol	C1S	P09871	CHEMBL3913	0.101614	20 / 0
Phytone	C3AR1	Q16581	CHEMBL4761	0.112042	26 / 0
Phytone	C5AR1	P21730	CHEMBL2373	0.112042	27 / 0
(1R,2R,4R)-Trihydroxy-p-menthane	CA1	P00915	CHEMBL261	0.071787	11 / 3
Caffeic acid	CA12	O43570	CHEMBL3242	0.729303	37 / 0
Caffeic acid	CA13	Q8N1Q1	CHEMBL3912	0.08057	9 / 0
Caffeic acid	CA14	Q9ULX7	CHEMBL3510	0.729303	14 / 0
(1R,2R,4R)-Trihydroxy-p-menthane	CA2	P00918	CHEMBL205	0.08057	129 / 0

Caffeic acid	CA3	P07451	CHEMBL2885	0.212409	5 / 0
Carvone Hydrate	CA4	P22748	CHEMBL3729	0.116739	11 / 6
Caffeic acid	CA5A	P35218	CHEMBL4789	0.729303	7 / 0
Caffeic acid	CA5B	Q9Y2D0	CHEMBL3969	0.729303	7 / 0
Caffeic acid	CA6	P23280	CHEMBL3025	0.729303	3 / 14
Caffeic acid	CA7	P43166	CHEMBL2326	0.729303	30 / 18
Caffeic acid	CA9	Q16790	CHEMBL3594	0.729303	42 / 0
Arachic acid	CACNA2D1	P54289	CHEMBL1919	0.111502	0 / 4
Chrysin	CALM1	P62158	CHEMBL6093	0.135616	0 / 1
Apigenin	CAMK2B	Q13554	CHEMBL4121	0.108771	2 / 2
Rosmarinic acid	CAMKK2	Q96RR4	CHEMBL5284	0.10934	1 / 0
Methyl caffeate	CBFB	Q13951	CHEMBL1615386	0.031227	1 / 0
Miscanthoside	CBR1	P16152	CHEMBL5586	0.106543	0 / 1
Apigenin	CCNB3	Q8WWL7	CHEMBL2094127	1	13 / 0
	CDK1	P06493			
	CCNB1	P14635			
	CCNB2	O95067			
5Z-Dodecenyl Acetate	CCNE1	P24864	CHEMBL1907605	0.060425	53 / 0
	CDK2	P24941			
Rosmarinic acid ethyl ester	CCNE1	P24864	CHEMBL3038471	0.115737	20 / 0
	CDK3	Q00526			
Phytone	CCR1	P32246	CHEMBL2413	0.112042	89 / 0
Phytone	CCR2	P41597	CHEMBL4015	0.112042	6 / 0
Geranyl acetate	CCR4	P51679	CHEMBL2414	0.031227	21 / 0
Phytone	CCR5	P51681	CHEMBL274	0.112042	0 / 1
Terpinyl Acetate	CCR8	P51685	CHEMBL4596	0.031227	12 / 0
Cosmosiine	CD38	P28907	CHEMBL4660	0.118883	0 / 3
Carvone Hydrate	CD81	P60033	CHEMBL1075180	0.125076	0 / 1
Carvone Hydrate	CDC25A	P30304	CHEMBL3775	0.125076	4 / 13
(1R,2R,4R)-Trihydroxy-p-menthane	CDC25B	P30305	CHEMBL4804	0.08057	15 / 11
Perillyl acetate	CDC25C	P30307	CHEMBL2378	0.031227	0 / 4
Arachic acid	CDC45	O75419	CHEMBL3040	0.111502	0 / 1
5Z-Dodecenyl Acetate	CDC7	O00311	CHEMBL5443	0.060425	2 / 0
Icariside E4	CDK1	P06493	CHEMBL308	0.103187	12 / 0
5Z-Dodecenyl Acetate	CDK19	Q9BWU1	CHEMBL6002	0.060425	1 / 0
Rosmarinic acid methyl ester	CDK2	P24941	CHEMBL301	0.113286	32 / 0
Rosmarinic acid ethyl ester	CDK2	P24941	CHEMBL2094128	0.115737	8 / 0
	CCNA1	P78396			
	CCNA2	P20248			
Rosmarinic acid methyl ester	CDK4	P11802	CHEMBL331	0.113286	53 / 0
Geranyl acetate	CDK5	Q00535	CHEMBL4036	0.031227	20 / 0
Carvone Hydrate	CDK5R1	Q15078	CHEMBL1907600	0.125076	24 / 0
	CDK5	Q00535			
Apigenin	CDK6	Q00534	CHEMBL2508	1	4 / 4

5Z-Dodecenyl Acetate	CDK8	P49336	CHEMBL5719	0.060425	27 / 0
Terpinyl Acetate	CEL	P19835	CHEMBL3219	0.031227	4 / 0
Rosmarinic acid ethyl ester	CELA1	Q9UNI1	CHEMBL3000	0.115737	2 / 0
Carvone Hydrate	CES1	P23141	CHEMBL2265	0.125076	0 / 32
Carvone Hydrate	CES2	O00748	CHEMBL3180	0.133391	19 / 12
Apigenin	CFTR	P13569	CHEMBL4051	1	2 / 1
Rosmarinic acid ethyl ester	CHEK1	O14757	CHEMBL4630	0.115737	221 / 0
Phytone	CHRM1	P11229	CHEMBL216	0.112042	28 / 0
Vinyl Amyl Carbinol	CHRM2	P08172	CHEMBL211	0.023833	0 / 1
Phytone	CHRM3	P20309	CHEMBL245	0.112042	29 / 0
Vinyl Amyl Carbinol	CHRM4	P08173	CHEMBL1821	0.023833	12 / 0
3,7-Dimethylocta-2,6-dienyl propanoate	CHRM5	P08912	CHEMBL2035	0.042894	14 / 0
Terpinyl Acetate	CHRNA4	P43681	CHEMBL1882	0.031227	1 / 0
1-Carvone	CHRNA4<br>CHRNB2	P43681<br>P17787	CHEMBL1907589	0.043919	7 / 0
Arachic acid	CHRNA7	P36544	CHEMBL2492	0.111502	0 / 2
Geranyl acetate	CHUK	O15111	CHEMBL3476	0.031227	8 / 0
Methyl caffeate	CISD1	Q9NZ45	CHEMBL1795168	0.031227	2 / 0
5Z-Dodecenyl Acetate	CIT	O14578	CHEMBL5579	0.060425	2 / 0
(+/-)-Eriodictyol	CLK1	P49759	CHEMBL4224	0.097875	10 / 0
5Z-Dodecenyl Acetate	CLK4	Q9HAZ1	CHEMBL4203	0.060425	70 / 0
Phytone	CNR1	P21554	CHEMBL218	0.112042	723 / 0
Phytone	CNR2	P34972	CHEMBL253	0.112042	1 / 1
3,4-Dihydroxy-5-methoxybenzaldehyde	COMT	P21964	CHEMBL2023	0.116739	0 / 5
Terpinyl Acetate	CREBBP	Q92793	CHEMBL5747	0.031227	1 / 0
5Z-Dodecenyl Acetate	CSF1R	P07333	CHEMBL1844	0.060425	135 / 0
5Z-Dodecenyl Acetate	CSNK1A1	P48729	CHEMBL2793	0.060425	1 / 0
5Z-Dodecenyl Acetate	CSNK1D	P48730	CHEMBL2828	0.060425	2 / 0
3,7-Dimethylocta-2,6-dienyl propanoate	CSNK1E	P49674	CHEMBL4937	0.042894	1 / 0
Apigenin	CSNK2A1	P68400	CHEMBL3629	1	2 / 2
Caffeic acid	CTBP2	P56545	CHEMBL3797016	0.071787	10 / 0
Terpinyl Acetate	CTRB1	P17538	CHEMBL4796	0.031227	4 / 0
Terpinyl Acetate	CTRC	Q99895	CHEMBL2386	0.04147	4 / 3
(+/-)-Eriodictyol	CTSB	P07858	CHEMBL4072	0.097875	40 / 0
D-Myrtenal	CTSD	P07339	CHEMBL2581	0.043919	0 / 1
Geranyl acetate	CTSH	P09668	CHEMBL2225	0.031227	7 / 0
5Z-Dodecenyl Acetate	CTSK	P43235	CHEMBL268	0.060425	95 / 0
Phytone	CTSL	P07711	CHEMBL3837	0.112042	62 / 0
3,7-Dimethylocta-2,6-dienyl propanoate	CTSS	P25774	CHEMBL2954	0.042894	155 / 0
2- Dodecenal	CTSV	O60911	CHEMBL3272	0.071787	8 / 0
Decanoic acid	CXCL8	P10145	CHEMBL2157	0.141787	12 / 0
Apigenin	CXCR1	P25024	CHEMBL4029	0.108771	2 / 1
Cyclohexane	CXCR3	P49682	CHEMBL4441	0.042894	2 / 0
Carvone Hydrate	CYP11B1	P15538	CHEMBL1908	0.125076	178 / 0

Carvone Hydrate	CYP11B2	P19099	CHEMBL2722	0.125076	221 / 0
Carvone Hydrate	CYP17A1	P05093	CHEMBL3522	0.133391	81 / 9
Carvone Hydrate	CYP19A1	P11511	CHEMBL1978	0.150098	0 / 163
Chrysin	CYP1A1	P04798	CHEMBL2231	0.12775	0 / 7
Caffeic acid	CYP1A2	P05177	CHEMBL3356	0.071787	2 / 5
Miscanthoside	CYP1B1	Q16678	CHEMBL4878	0.106543	0 / 6
Palmitic Acid	CYP26A1	O43174	CHEMBL5141	0.053556	7 / 0
Palmitic Acid	CYP26B1	Q9NR63	CHEMBL3713687	0.053556	9 / 0
2- Dodecenal	CYP27A1	Q02318	CHEMBL5992	0.071787	3 / 0
Geranyl acetate	CYP2A6	P11509	CHEMBL5282	0.031227	21 / 0
Caffeic acid	CYP2C19	P33261	CHEMBL3622	0.071787	0 / 1
Caffeic acid	CYP2C9	P11712	CHEMBL3397	0.071787	0 / 3
Caffeic acid	CYP3A4	P08684	CHEMBL340	0.071787	0 / 3
Carvone Hydrate	CYP51A1	Q16850	CHEMBL3849	0.125076	2 / 0
Coleonolic acid	CYSLTR1	Q9Y271	CHEMBL1798	0.120226	19 / 0
Methyl caffeate	DAO	P14920	CHEMBL5485	0.031227	34 / 0
Apigenin	DAPK1	P53355	CHEMBL2558	0.116965	2 / 2
3,7-Dimethylocta-2,6-dienyl propanoate	DAPK3	O43293	CHEMBL2468	0.042894	12 / 0
Decyl acetate	DCK	P27707	CHEMBL2447	0.031227	9 / 0
3,7-Dimethylocta-2,6-dienyl propanoate	DCTPP1	Q9H773	CHEMBL3769292	0.042894	7 / 0
5Z-Dodecenyl Acetate	DDR1	Q08345	CHEMBL5319	0.060425	6 / 0
5Z-Dodecenyl Acetate	DGAT1	O75907	CHEMBL6009	0.060425	20 / 0
β-sitosterol	DHCR7	Q9UBM7	CHEMBL2169735	0.106166	3 / 5
Rosmarinic acid	DHODH	Q02127	CHEMBL1966	0.10934	16 / 0
Rosmarinic acid ethyl ester	DNM1	Q05193	CHEMBL4958	0.115737	13 / 0
Coleonolic acid	DNTT	P04053	CHEMBL4810	0.120226	0 / 3
Rosmarinic acid methyl ester	DRD1	P21728	CHEMBL2056	0.113286	3 / 0
Carvone Hydrate	DRD2	P14416	CHEMBL217	0.125076	11 / 0
Amentoflavone	DRD3	P35462	CHEMBL234	1	3 / 0
Carvone Hydrate	DRD4	P21917	CHEMBL219	0.125076	4 / 0
3,4-Dihydroxy-5-methoxybenzaldehyde	DUSP1	P28562	CHEMBL6026	0.116739	1 / 0
Coleonolic acid	DUSP3	P51452	CHEMBL2635	0.120226	4 / 0
Rosmarinic acid ethyl ester	DUT	P33316	CHEMBL5203	0.115737	7 / 0
(+/-)-Eriodictyol	DYRK1A	Q13627	CHEMBL2292	0.097875	54 / 0
(+/-)-Eriodictyol	DYRK1B	Q9Y463	CHEMBL5543	0.097875	26 / 0
2α,19α-Dihydroxyursolic acid	EDNRA	P25101	CHEMBL252	0.110612	102 / 0
2α,19α-Dihydroxyursolic acid	EDNRB	P24530	CHEMBL1785	0.110612	53 / 0
Caffeic acid	EGFR	P00533	CHEMBL203	0.071787	229 / 0
Rosmarinic acid methyl ester	EIF2AK2	P19525	CHEMBL5785	0.113286	6 / 0
Miscanthoside	EIF4A1	P60842	CHEMBL2052028	0.106543	0 / 29
Rosmarinic acid	EIF4H	Q15056	CHEMBL1293274	0.10934	1 / 0
Caffeic acid	ELANE	P08246	CHEMBL248	0.071787	5 / 2
Rosmarinic acid ethyl ester	EP300	Q09472	CHEMBL3784	0.115737	9 / 0
Terpinyl Acetate	EPAS1	Q99814	CHEMBL1744522	0.031227	20 / 1

Rosmarinic acid methyl ester	EPHB4	P54760	CHEMBL5147	0.113286	39 / 0
Phytone	EPHX1	P07099	CHEMBL1968	0.112042	42 / 0
Phytone	EPHX2	P34913	CHEMBL2409	0.112042	6 / 0
Caffeic acid	ERBB2	P04626	CHEMBL1824	0.071787	9 / 3
3,4-Dihydroxy-5-methoxybenzaldehyde	ERN1	O75460	CHEMBL1163101	0.150098	65 / 0
(1R,2R,4R)-Trihydroxy-p-menthane	ESR1	P03372	CHEMBL206	0.08057	0 / 30
Carvone Hydrate	ESR2	Q92731	CHEMBL242	0.116739	0 / 10
Rosmarinic acid methyl ester	ESRRA	P11474	CHEMBL3429	0.113286	18 / 0
Rosmarinic acid methyl ester	ESRRB	O95718	CHEMBL3751	0.113286	18 / 0
Thymol	ESRRG	P62508	CHEMBL4245	0.043919	0 / 1
Cosmosiine	F10	P00742	CHEMBL244	0.118883	17 / 0
PubChem CID 21637755	F2	P00734	CHEMBL204	0.087714	10 / 7
5Z-Dodecenyl Acetate	F2R	P25116	CHEMBL3974	0.060425	103 / 0
Caffeic acid	F3	P13726	CHEMBL4081	0.071787	0 / 9
2α,19α-Dihydroxyursolic acid	FAAH	O00519	CHEMBL2243	0.110612	9 / 9
Carvone Hydrate	FABP1	P07148	CHEMBL5421	0.125076	0 / 2
Arachic acid	FABP2	P12104	CHEMBL4879	0.340526	2 / 1
Carvone Hydrate	FABP3	P05413	CHEMBL3344	0.125076	0 / 4
Carvone Hydrate	FABP4	P15090	CHEMBL2083	0.125076	0 / 3
Carvone Hydrate	FABP5	Q01469	CHEMBL3674	0.125076	0 / 1
2α,19α-Dihydroxyursolic acid	FDFT1	P37268	CHEMBL3338	0.110612	0 / 1
Arachic acid	FFAR1	O14842	CHEMBL4422	0.26156	58 / 3
Palmitic Acid	FFAR4	Q5NUL3	CHEMBL5339	0.053556	15 / 0
(+/-)-Eriodictyol	FGFR1	P11362	CHEMBL3650	0.097875	5 / 0
Carvone Hydrate	FKBP1A	P62942	CHEMBL1902	0.116739	2 / 0
5Z-Dodecenyl Acetate	FLT1	P17948	CHEMBL1868	0.060425	124 / 0
Rosmarinic acid methyl ester	FLT3	P36888	CHEMBL1974	0.113286	11 / 0
5Z-Dodecenyl Acetate	FLT4	P35916	CHEMBL1955	0.060425	34 / 0
	FNTA	P49354			
2α,19α-Dihydroxyursolic acid			CHEMBL2094108	0.110612	37 / 9
	FNTB	P49356			
5Z-Dodecenyl Acetate	FRK	P42685	CHEMBL4223	0.060425	4 / 0
(+/-)-Eriodictyol	FUT4	P22083	CHEMBL4996	0.097875	0 / 1
3,4-Dihydroxy-5-methoxybenzaldehyde	FUT7	Q11130	CHEMBL3596077	0.116739	0 / 2
Caffeic acid	FYN	P06241	CHEMBL1841	0.071787	0 / 2
Carvone Hydrate	G6PD	P11413	CHEMBL5347	0.125076	0 / 2
Arachic acid	GABBR1	Q9UBS5	CHEMBL2064	0.127303	0 / 4
Geranyl acetate	GABRA1	P14867	CHEMBL1962	0.031227	24 / 0
	GABRA1	P14867			
Amentoflavone	GABRB2	P47870	CHEMBL2095172	1	5 / 3
	GABRG2	P18507			
	GABRA2	P47869			
Arachic acid	GABRB2	P47870	CHEMBL2111413	0.119404	3 / 0
	GABRG2	P18507			
Geranyl acetate	GABRA2	P47869	CHEMBL2094130	0.031227	21 / 0

	GABRB3	P28472			
	GABRG2	P18507			
Geranyl acetate	GABRA5	P31644	CHEMBL5112	0.031227	21 / 0
	GABRB3	P28472			
Geranyl acetate	GABRA3	P34903	CHEMBL2094120	0.031227	27 / 0
	GABRG2	P18507			
	GABRB3	P28472			
Geranyl acetate	GABRG2	P18507	CHEMBL2094121	0.031227	22 / 0
	GABRA1	P14867			
	GABRB3	P28472			
Geranyl acetate	GABRG2	P18507	CHEMBL2094122	0.031227	55 / 0
	GABRA5	P31644			
	GABRG2	P18507			
2- Dodecenal	GABRB3	P28472	CHEMBL2095190	0.071787	6 / 0
	GABRA6	Q16445			
3,7-Dimethylocta-2,6-dienyl propanoate	GAK	O14976	CHEMBL4355	0.042894	3 / 0
5Z-Dodecenyl Acetate	GCGR	P47871	CHEMBL1985	0.060425	3 / 0
5Z-Dodecenyl Acetate	GCK	P35557	CHEMBL3820	0.060425	15 / 0
PubChem CID 21637755	GLI1	P08151	CHEMBL5461	0.079146	0 / 2
Gallocatechol	GLI2	P10070	CHEMBL5119	0.101614	0 / 1
Apigenin	GLO1	Q04760	CHEMBL2424	0.370888	3 / 4
β-sitosterol	GLRA1	P23415	CHEMBL5845	0.106166	1 / 1
Carvone Hydrate	GLUL	P15104	CHEMBL4612	0.116739	0 / 1
Carvone Hydrate	GPBAR1	Q8TDU6	CHEMBL5409	0.116739	0 / 16
Gallic acid	GPR35	Q9HC97	CHEMBL1293267	0.116739	1 / 0
Terpinyl Acetate	GPR55	Q9Y2T6	CHEMBL1075322	0.031227	2 / 0
Phytone	GPR88	Q9GZN0	CHEMBL3399910	0.112042	2 / 0
Decyl acetate	GRIA2	P42262	CHEMBL4016	0.031227	9 / 0
Decyl acetate	GRIA4	P48058	CHEMBL3190	0.031227	2 / 0
Oleanolic acid	GRIK1	P39086	CHEMBL1918	0.100634	0 / 26
Maslinic acid	GRIK2	Q13002	CHEMBL3683	0.120226	0 / 6
5Z-Dodecenyl Acetate	GRIN2B	Q13224	CHEMBL1904	0.060425	18 / 0
Rosmarinic acid ethyl ester	GRK2	P25098	CHEMBL4079	0.115737	12 / 0
Apigenin	GRK6	P43250	CHEMBL6144	0.329868	3 / 0
Phytone	GRM1	Q13255	CHEMBL3772	0.112042	60 / 0
5Z-Dodecenyl Acetate	GRM2	Q14416	CHEMBL5137	0.060425	11 / 0
Geranyl acetate	GRM4	Q14833	CHEMBL2736	0.031227	32 / 0
Arachic acid	GRM5	P41594	CHEMBL3227	0.111502	225 / 0
Apigenin	GSK3B	P49841	CHEMBL262	1	19 / 1
Carvone Hydrate	GSR	P00390	CHEMBL2755	0.125076	2 / 0
Arachic acid	GSTK1	Q9Y2Q3	CHEMBL4491	0.111502	0 / 1
Rosmarinic acid methyl ester	GUSB	P08236	CHEMBL2728	0.113286	15 / 0
Carvone Hydrate	HAO1	Q9UJM8	CHEMBL4229	0.116739	7 / 1
Caffeic acid	HCAR2	Q8TDS4	CHEMBL3785	0.08057	0 / 1

Rosmarinic acid ethyl ester	HDAC1	Q13547	CHEMBL325	0.115737	537 / 0
Rosmarinic acid methyl ester	HDAC2	Q92769	CHEMBL1937	0.113286	186 / 0
Rosmarinic acid ethyl ester	HDAC3	O15379	CHEMBL1829	0.115737	198 / 0
Rosmarinic acid ethyl ester	HDAC6	Q9UBN7	CHEMBL1865	0.115737	354 / 0
Rosmarinic acid methyl ester	HDAC8	Q9BY41	CHEMBL3192	0.113286	216 / 0
(+/-)-Eriodictyol	HIF1A	Q16665	CHEMBL4261	0.097875	0 / 2
2α,19α-Dihydroxyursolic acid	HMGCR	P04035	CHEMBL402	0.110612	0 / 37
Carvone Hydrate	HMOX1	P09601	CHEMBL2823	0.116739	7 / 0
Decyl acetate	HPGD	P15428	CHEMBL1293255	0.031227	16 / 0
Geranyl acetate	HPRT1	P00492	CHEMBL2360	0.031227	1 / 0
CAS 1038743-23-5	HRAS	P01112	CHEMBL2167	0.055648	1 / 0
5Z-Dodecenyl Acetate	HRH1	P35367	CHEMBL231	0.060425	30 / 0
5Z-Dodecenyl Acetate	HRH3	Q9Y5N1	CHEMBL264	0.060425	70 / 0
5Z-Dodecenyl Acetate	HRH4	Q9H3N8	CHEMBL3759	0.060425	36 / 0
Carvone Hydrate	HSD11B1	P28845	CHEMBL4235	0.125076	229 / 16
Carvone Hydrate	HSD11B2	P80365	CHEMBL3746	0.125076	0 / 18
Miscanthoside	HSD17B1	P14061	CHEMBL3181	0.106543	0 / 1
Carvone Hydrate	HSD17B2	P37059	CHEMBL2789	0.125076	37 / 0
Arachic acid	HSD17B3	P37058	CHEMBL4234	0.127303	19 / 0
Phytone	HSD3B1	P14060	CHEMBL1958	0.112042	10 / 0
Phytone	HSP90AA1	P07900	CHEMBL3880	0.112042	14 / 0
Rosmarinic acid methyl ester	HSP90AB1	P08238	CHEMBL4303	0.113286	69 / 0
5Z-Dodecenyl Acetate	HTR2A	P28223	CHEMBL224	0.060425	20 / 0
Palmitic Acid	HTR2B	P41595	CHEMBL1833	0.053556	8 / 0
Amentoflavone	HTR2C	P28335	CHEMBL225	1	30 / 0
1-Carvone	HTR7	P34969	CHEMBL3155	0.043919	1 / 0
Geranyl acetate	HTT	P42858	CHEMBL5514	0.031227	4 / 0
Terpinyl Acetate	ICAM1	P05362	CHEMBL3070	0.031227	3 / 0
Phytone	ICMT	O60725	CHEMBL4699	0.112042	45 / 0
Geranyl acetate	IDH1	O75874	CHEMBL2007625	0.031227	134 / 0
Carvone Hydrate	IDO1	P14902	CHEMBL4685	0.116739	16 / 0
Gallic acid	IGF1R	P08069	CHEMBL1957	0.125076	0 / 3
Rosmarinic acid	IGFBP3	P17936	CHEMBL3997	0.10934	28 / 0
Rosmarinic acid	IGFBP5	P24593	CHEMBL2665	0.10934	7 / 0
Chrysin	IKBKB	O14920	CHEMBL1991	0.198444	29 / 0
Perillyl acetate	IL1B	P01584	CHEMBL1909490	0.031227	0 / 2
Cosmosiine	IL2	P60568	CHEMBL5880	0.295838	0 / 4
Carvone Hydrate	IL6	P05231	CHEMBL1795129	0.116739	0 / 1
Rosmarinic acid ethyl ester	IMPDH1	P20839	CHEMBL1822	0.115737	14 / 0
5Z-Dodecenyl Acetate	IMPDH2	P12268	CHEMBL2002	0.060425	12 / 0
Rosmarinic acid methyl ester	INSR	P06213	CHEMBL1981	0.113286	10 / 0
Carvone Hydrate	ITGAL	P20701	CHEMBL1803	0.116739	0 / 4
Palmitic Acid	ITGAL<br>ICAM1	P20701<br>P05362	CHEMBL2096661	0.053556	6 / 0

	ITGB2	P05107			
2α,19α-Dihydroxyursolic acid	ITGB1 ITGA4	P05556<br>P13612	CHEMBL1907599	0.110612	246 / 0
5Z-Dodecenyl Acetate	JAK1	P23458	CHEMBL2835	0.060425	65 / 0
5Z-Dodecenyl Acetate	JAK2	O60674	CHEMBL2971	0.060425	99 / 0
Vinyl Amyl Carbinol	JAK3	P52333	CHEMBL2148	0.023833	43 / 0
PubChem CID 21637755	JUN	P05412	CHEMBL4977	0.079146	2 / 5
Methyl geranate	KAT2B	Q92831	CHEMBL5500	0.071787	5 / 0
Vinyl Amyl Carbinol	KCNA5	P22460	CHEMBL4306	0.023833	9 / 0
Rosmarinic acid methyl ester	KCNH2	Q12809	CHEMBL240	0.113286	9 / 0
5Z-Dodecenyl Acetate	KCNJ11<br>ABCC9	Q14654<br>O60706	CHEMBL2095198	0.060425	9 / 0
3-Cyclohexene-1-methanol	KCNJ5<br>KCNJ3	P48544<br>P48549	CHEMBL3038488	0.031227	3 / 0
Terpinyl Acetate	KCNK2	O95069	CHEMBL2321615	0.031227	1 / 0
Perillyl acetate	KCNMA1	Q12791	CHEMBL4304	0.031227	5 / 0
Arachic acid	KDM2A	Q9Y2K7	CHEMBL1938210	0.127303	3 / 1
Caffeic acid	KDM4A	O75164	CHEMBL5896	0.071787	4 / 0
Caffeic acid	KDM4E	B2RXH2	CHEMBL1293226	0.071787	4 / 0
Arachic acid	KDM5C	P41229	CHEMBL2163176	0.127303	4 / 1
Phytone	KDR	P35968	CHEMBL279	0.112042	66 / 0
3-Cyclohexene-1-methanol	KIF11	P52732	CHEMBL4581	0.031227	3 / 0
3,7-Dimethylocta-2,6-dienyl propanoate	KIF20A	O95235	CHEMBL2021753	0.042894	4 / 0
5Z-Dodecenyl Acetate	KIT	P10721	CHEMBL1936	0.060425	130 / 0
Luteolin 7-(6''-ferulylglucoside)	KLK1	P06870	CHEMBL2319	0.055648	1 / 0
Luteolin 7-(6''-ferulylglucoside)	KLK2	P20151	CHEMBL2442	0.055648	1 / 0
Methyl geranate	LAP3	P28838	CHEMBL3965	0.071787	0 / 1
Caffeic acid	LCK	P06239	CHEMBL258	0.071787	8 / 6
Gallic acid	LDHA	P00338	CHEMBL4835	0.125076	0 / 14
Gallic acid	LDHB	P07195	CHEMBL4940	0.125076	0 / 11
Phytone	LDLR	P01130	CHEMBL3311	0.112042	10 / 0
5Z-Dodecenyl Acetate	LIMK2	P53671	CHEMBL5932	0.060425	66 / 0
Geranyl acetate	LIPE	Q05469	CHEMBL3590	0.031227	20 / 0
Carvone Hydrate	LRRK2	Q5S007	CHEMBL1075104	0.125076	9 / 0
Caryophyllene oxide	LSS	P48449	CHEMBL3593	0.060425	0 / 8
Arachic acid	LTA4H	P09960	CHEMBL4618	0.111502	0 / 1
2α,19α-Dihydroxyursolic acid	LTB4R	Q15722	CHEMBL3911	0.110612	0 / 4
Daucosterol	MAOA	P21397	CHEMBL1951	0.074392	125 / 1
Caffeic acid	MAOB	P27338	CHEMBL2039	0.071787	239 / 0
Rotungenoside	MAP2K1	Q02750	CHEMBL3587	0.035898	47 / 0
Rosmarinic acid ethyl ester	MAP2K2	P36507	CHEMBL2964	0.115737	5 / 0
5Z-Dodecenyl Acetate	MAP3K19	Q56UN5	CHEMBL6191	0.060425	5 / 0
5Z-Dodecenyl Acetate	MAP3K20	Q9NYL2	CHEMBL3886	0.060425	2 / 0
Caffeic acid	MAPK1	P28482	CHEMBL4040	0.071787	7 / 1

5Z-Dodecenyl Acetate	MAPK10	P53779	CHEMBL2637	0.060425	187 / 0
Phytone	MAPK14	Q16539	CHEMBL260	0.112042	24 / 0
Carvone Hydrate	MAPK3	P27361	CHEMBL3385	0.133391	0 / 1
Rotungenoside	MAPK8	P45983	CHEMBL2276	0.035898	35 / 0
Rotungenoside	MAPK9	P45984	CHEMBL4179	0.035898	15 / 0
Gallocatechol	MAPT	P10636	CHEMBL1293224	0.101614	4 / 1
Methyl caffeate	MB	P02144	CHEMBL2406892	0.031227	4 / 0
5Z-Dodecenyl Acetate	MCL1	Q07820	CHEMBL4361	0.060425	4 / 0
Phytone	MDM2	Q00987	CHEMBL5023	0.112042	260 / 0
3,7-Dimethylocta-2,6-dienyl propanoate	MEN1	O00255	CHEMBL1615381	0.042894	2 / 0
Rotungenoside	MERTK	Q12866	CHEMBL5331	0.035898	22 / 0
Daucosterol	MET	P08581	CHEMBL3717	0.074392	44 / 22
Neryl Acetate	METAP1	P53582	CHEMBL2474	0.031227	2 / 0
Rosmarinic acid	METAP2	P50579	CHEMBL3922	0.10934	11 / 0
Phytone	MGLL	Q99685	CHEMBL4191	0.112042	41 / 0
Carvone Hydrate	MIF	P14174	CHEMBL2085	0.116739	18 / 1
2α,19α-Dihydroxyursolic acid	MME	P08473	CHEMBL1944	0.110612	118 / 0
Caffeic acid	MMP1	P03956	CHEMBL332	0.729303	43 / 27
Miscanthoside	MMP12	P39900	CHEMBL4393	0.106543	3 / 3
Miscanthoside	MMP13	P45452	CHEMBL280	0.106543	1 / 1
Rosmarinic acid	MMP14	P50281	CHEMBL3869	0.10934	118 / 0
Rosmarinic acid methyl ester	MMP15	P51511	CHEMBL2963	0.113286	12 / 0
Rosmarinic acid methyl ester	MMP16	P51512	CHEMBL2200	0.113286	24 / 0
Caffeic acid	MMP2	P08253	CHEMBL333	0.729303	38 / 27
Rosmarinic acid methyl ester	MMP26	Q9NRE1	CHEMBL4707	0.113286	10 / 0
2α,19α-Dihydroxyursolic acid	MMP3	P08254	CHEMBL283	0.110612	40 / 0
Rosmarinic acid ethyl ester	MMP7	P09237	CHEMBL4073	0.115737	107 / 0
CAS 1038743-23-5	MMP8	P22894	CHEMBL4588	0.055648	10 / 0
Caffeic acid	MMP9	P14780	CHEMBL321	0.729303	42 / 25
Apigenin	MPG	P29372	CHEMBL3396943	0.116965	1 / 1
3,4-Dihydroxy-5-methoxybenzaldehyde	MPO	P05164	CHEMBL2439	0.116739	9 / 0
Phytone	MTNR1A	P48039	CHEMBL1945	0.112042	156 / 0
Phytone	MTNR1B	P49286	CHEMBL1946	0.112042	126 / 0
PubChem CID 21637755	MTOR	P42345	CHEMBL2842	0.079146	98 / 0
Chrysin	MYLK	Q15746	CHEMBL2428	0.119895	0 / 1
5Z-Dodecenyl Acetate	NAAA	Q02083	CHEMBL4349	0.060425	30 / 0
Apigenin	NAE1	Q13564	CHEMBL2016431	0.141522	0 / 1
Phytone	NAMPT	P43490	CHEMBL1744525	0.112042	113 / 0
Vanillin	NAT1	P18440	CHEMBL5101	0.043919	3 / 0
Apigenin	NEK2	P51955	CHEMBL3835	0.108771	1 / 2
Apigenin	NEK6	Q9HC98	CHEMBL4309	0.108771	1 / 2
Caffeic acid	NFE2L2	Q16236	CHEMBL1075094	0.071787	0 / 4
Caffeic acid	NGFR	P08138	CHEMBL4762	0.071787	2 / 0
Terpinyl Acetate	NLRP3	Q96P20	CHEMBL1741208	0.031227	0 / 1

2- Dodecenal	NMT1	P30419	CHEMBL2593	0.071787	2 / 0
5Z-Dodecenyl Acetate	NOD1	Q9Y239	CHEMBL1293222	0.060425	15 / 0
Perillyl acetate	NOS1	P29475	CHEMBL3568	0.031227	19 / 0
Carvone Hydrate	NOS2	P35228	CHEMBL4481	0.133391	9 / 14
m-alpha-Terpineol, acetate	NOS3	P29474	CHEMBL4803	0.031227	5 / 0
Cosmosiine	NOX4	Q9NPH5	CHEMBL1250375	0.118883	1 / 7
Carvone Hydrate	NPC1L1	Q9UHC9	CHEMBL2027	0.150098	0 / 15
Phytone	NPY5R	Q15761	CHEMBL4561	0.112042	58 / 0
1-Carvone	NQO1	P15559	CHEMBL3623	0.391123	1 / 0
Caffeic acid	NQO2	P16083	CHEMBL3959	0.071787	0 / 17
Palmitic Acid	NR0B2	Q15466	CHEMBL5603	0.053556	1 / 0
β-sitosterol	NR1H2	P55055	CHEMBL4093	0.106166	0 / 1
Carvone Hydrate	NR1H3	Q13133	CHEMBL2808	0.125076	0 / 17
(1R,2R,4R)-Trihydroxy-p-menthane	NR1H4	Q96RI1	CHEMBL2047	0.08937	0 / 12
Carvone Hydrate	NR1I2	O75469	CHEMBL3401	0.125076	1 / 0
Carvone Hydrate	NR1I3	Q14994	CHEMBL5503	0.125076	0 / 2
Carvone Hydrate	NR3C1	P04150	CHEMBL2034	0.158398	0 / 11
Carvone Hydrate	NR3C2	P08235	CHEMBL1994	0.141787	0 / 7
Rosmarinic acid ethyl ester	NR4A1	P22736	CHEMBL1293229	0.115737	2 / 0
Chrysin	NTRK2	Q16620	CHEMBL4898	0.198444	1 / 1
Kaempferol	NUAK1	O60285	CHEMBL5784	0.402643	1 / 2
3-Cyclohexene-1-methanol	NUDT1	P36639	CHEMBL3708265	0.031227	4 / 0
(+/-)-Eriodictyol	ODC1	P11926	CHEMBL1869	0.097875	0 / 5
Phytone	OPRD1	P41143	CHEMBL236	0.112042	5 / 0
Vinyl Amyl Carbinol	OPRK1	P41145	CHEMBL237	0.023833	7 / 0
Phytone	OPRM1	P35372	CHEMBL233	0.112042	8 / 0
Phytone	OXTR	P30559	CHEMBL2049	0.112042	138 / 0
Phytone	P2RX7	Q99572	CHEMBL4805	0.112042	104 / 0
Rosmarinic acid	PABPC1	P11940	CHEMBL1293286	0.10934	4 / 0
Phytone	PAOX	Q6QHF9	CHEMBL2105	0.112042	0 / 1
Phytone	PARP1	P09874	CHEMBL3105	0.112042	24 / 0
3,7-Dimethylocta-2,6-dienyl propanoate	PDE10A	Q9Y233	CHEMBL4409	0.042894	62 / 0
3,7-Dimethylocta-2,6-dienyl propanoate	PDE4A	P27815	CHEMBL254	0.042894	43 / 0
2α,19α-Dihydroxyursolic acid	PDE4D	Q08499	CHEMBL288	0.128067	16 / 9
2α,19α-Dihydroxyursolic acid	PDE5A	O76074	CHEMBL1827	0.110612	8 / 0
3-Cyclohexene-1-methanol	PDE7A	Q13946	CHEMBL3012	0.031227	45 / 0
3,7-Dimethylocta-2,6-dienyl propanoate	PDE9A	O76083	CHEMBL3535	0.042894	1 / 0
5Z-Dodecenyl Acetate	PDGFRA	P16234	CHEMBL2007	0.060425	35 / 0
Decyl acetate	PDGFRA<br>PDGFRB	P16234<br>P09619	CHEMBL2095189	0.031227	10 / 0
5Z-Dodecenyl Acetate	PDGFRB	P09619	CHEMBL1913	0.060425	87 / 0
Rosmarinic acid ethyl ester	PDK1	Q15118	CHEMBL4766	0.115737	244 / 0
Decanoic acid	PDPK1	O15530	CHEMBL2534	0.116739	2 / 0
5Z-Dodecenyl Acetate	PFKFB3	Q16875	CHEMBL2331053	0.060425	159 / 0

Rosmarinic acid methyl ester	PGD	P52209	CHEMBL3404	0.113286	4 / 0
Amentoflavone	PGF	P49763	CHEMBL1697671	1	1 / 1
Decyl acetate	PGGT1B<br>FNTA	P53609<br>P49354	CHEMBL2095164	0.031227	24 / 0
Carvone Hydrate	PGR	P06401	CHEMBL208	0.150098	120 / 40
Arachic acid	PHF8	Q9UPP1	CHEMBL1938212	0.127303	0 / 1
Caffeic acid	PIK3CA	P42336	CHEMBL4005	0.071787	0 / 1
(1R,2R,4R)-Trihydroxy-p-menthane	PIK3CA<br>PIK3R1	P42336<br>P27986	CHEMBL2111367	0.071787	1 / 0
Caffeic acid	PIK3CB	P42338	CHEMBL3145	0.071787	0 / 1
Geranyl acetate	PIK3CD<br>PIK3R1	O00329<br>P27986	CHEMBL2111432	0.031227	2 / 0
Phytone	PIK3CG	P48736	CHEMBL3267	0.112042	64 / 0
Apigenin	PIK3R1	P27986	CHEMBL2506	0.108771	1 / 1
Phytone	PIM1	P11309	CHEMBL2147	0.112042	41 / 0
Phytone	PIM3	Q86V86	CHEMBL5407	0.112042	2 / 0
5Z-Dodecenyl Acetate	PKM	P14618	CHEMBL1075189	0.060425	32 / 0
Apigenin	PKN1	Q16512	CHEMBL3384	0.108771	2 / 3
Arachic acid	PLA2G10	O15496	CHEMBL4342	0.111502	3 / 0
Carvone Hydrate	PLA2G1B	P04054	CHEMBL4426	0.125076	0 / 1
Chrysin	PLA2G2A	P14555	CHEMBL3474	0.112042	3 / 6
2α,19α-Dihydroxyursolic acid	PLA2G4A	P47712	CHEMBL3816	0.110612	6 / 0
(+/-)-Eriodictyol	PLA2G5	P39877	CHEMBL4323	0.097875	0 / 3
Terpinyl Acetate	PLA2G6	O60733	CHEMBL3213	0.031227	3 / 0
Vanillin	PLAA	Q9Y263	CHEMBL6114	0.043919	10 / 0
Geranyl acetate	PLAU	P00749	CHEMBL3286	0.031227	3 / 0
Methyl caffeate	PLEC	Q15149	CHEMBL1293240	0.031227	1 / 0
Arachic acid	PLG	P00747	CHEMBL1801	0.111502	0 / 1
Apigenin	PLK1	P53350	CHEMBL3024	0.125143	2 / 3
Methyl caffeate	PNP	P00491	CHEMBL4338	0.031227	25 / 0
5Z-Dodecenyl Acetate	PNPLA2	Q96AD5	CHEMBL3822353	0.060425	1 / 0
(1R,2R,4R)-Trihydroxy-p-menthane	POLA1	P09884	CHEMBL1828	0.08937	0 / 1
Carvone Hydrate	POLB	P06746	CHEMBL2392	0.125076	0 / 4
5Z-Dodecenyl Acetate	PON1	P27169	CHEMBL3167	0.060425	1 / 0
2α,19α-Dihydroxyursolic acid	PPARA	Q07869	CHEMBL239	0.110612	0 / 11
Carvone Hydrate	PPARD	Q03181	CHEMBL3979	0.116739	0 / 8
2α,19α-Dihydroxyursolic acid	PPARG	P37231	CHEMBL235	0.110612	0 / 12
Bodinioside A	PPM1B	O75688	CHEMBL2845	0.041891	0 / 1
Daucosterol	PPP1CC	P36873	CHEMBL4438	0.074392	0 / 1
Bodinioside B	PPP2CA	P67775	CHEMBL4703	0.057784	0 / 5
Bodinioside A	PPP2R5A	Q15172	CHEMBL4763	0.041891	0 / 1
2α,19α-Dihydroxyursolic acid	PREP	P48147	CHEMBL3202	0.110612	0 / 2
Decyl acetate	PRF1	P14222	CHEMBL5480	0.031227	14 / 0
Luteolin 7-(6''-ferulylglucoside)	PRKACA	P17612	CHEMBL4101	0.055648	20 / 0

Rotungenoside	PRKCA	P17252	CHEMBL299	0.035898	4 / 0
Luteolin 7-(6''-ferulylglucoside)	PRKCB	P05771	CHEMBL3045	0.055648	107 / 0
Luteolin 7-(6''-ferulylglucoside)	PRKCD	Q05655	CHEMBL2996	0.055648	94 / 0
Luteolin 7-(6''-ferulylglucoside)	PRKCE	Q02156	CHEMBL3582	0.055648	91 / 0
Luteolin 7-(6''-ferulylglucoside)	PRKCG	P05129	CHEMBL2938	0.055648	77 / 0
Carvone Hydrate	PRKCH	P24723	CHEMBL3616	0.125076	0 / 1
Chrysin	PRKDC	P78527	CHEMBL3142	0.119895	6 / 0
Geranyl acetate	PRSS1	P07477	CHEMBL209	0.031227	8 / 5
Arachic acid	PSEN1	P49768	CHEMBL2473	0.111502	7 / 0
	PSEN2	P49810			
	PSENEN	Q9NZ42			
	NCSTN	Q92542			
Carvone Hydrate	APH1A	Q96BI3	CHEMBL2094135	0.116739	14 / 0
	PSEN1	P49768			
	APH1B	Q8WW43			
Phytone	PTAFR	P25105	CHEMBL250	0.112042	10 / 0
2α,19α-Dihydroxyursolic acid	PTGDR	Q13258	CHEMBL4427	0.110612	56 / 0
2α,19α-Dihydroxyursolic acid	PTGDR2	Q9Y5Y4	CHEMBL5071	0.110612	338 / 0
2α,19α-Dihydroxyursolic acid	PTGER1	P34995	CHEMBL1811	0.110612	28 / 14
2α,19α-Dihydroxyursolic acid	PTGER2	P43116	CHEMBL1881	0.110612	22 / 5
Coleonolic acid	PTGER3	P43115	CHEMBL3710	0.120226	29 / 3
2α,19α-Dihydroxyursolic acid	PTGER4	P35408	CHEMBL1836	0.110612	87 / 11
Carvone Hydrate	PTGES	O14684	CHEMBL5658	0.125076	1 / 12
2α,19α-Dihydroxyursolic acid	PTGFR	P43088	CHEMBL1987	0.110612	1 / 1
2α,19α-Dihydroxyursolic acid	PTGIR	P43119	CHEMBL1995	0.110612	6 / 9
Caffeic acid	PTGS1	P23219	CHEMBL221	0.071787	6 / 19
Carvone Hydrate	PTGS2	P35354	CHEMBL230	0.116739	108 / 7
Apigenin	PTK2	Q05397	CHEMBL2695	0.108771	1 / 2
5Z-Dodecenyl Acetate	PTK2B	Q14289	CHEMBL5469	0.060425	21 / 0
5Z-Dodecenyl Acetate	PTK6	Q13882	CHEMBL4601	0.060425	20 / 0
PubChem CID 21637755	PTPA	Q15257	CHEMBL2505	0.079146	0 / 3
Caffeic acid	PTPN1	P18031	CHEMBL335	0.729303	0 / 24
Carvone Hydrate	PTPN11	Q06124	CHEMBL3864	0.166646	0 / 1
Carvone Hydrate	PTPN2	P17706	CHEMBL3807	0.141787	0 / 15
Carvone Hydrate	PTPN6	P29350	CHEMBL3166	0.125076	0 / 1
Arachic acid	PTPRC	P08575	CHEMBL3243	0.111502	21 / 0
Carvone Hydrate	PTPRF	P10586	CHEMBL3521	0.125076	2 / 0
Apigenin	PTPRS	Q13332	CHEMBL2396508	0.411906	4 / 8
Rosmarinic acid ethyl ester	PTPsigma	Q9UM81	CHEMBL2146342	0.115737	2 / 0
Apigenin	PYGL	P06737	CHEMBL2568	0.108771	1 / 1
5Z-Dodecenyl Acetate	QPCT	Q16769	CHEMBL4508	0.060425	13 / 0
Rosmarinic acid ethyl ester	RAF1	P04049	CHEMBL1906	0.115737	8 / 0
2- Dodecenal	RAPGEF4	Q8WZA2	CHEMBL2029198	0.071787	3 / 0
Palmitic Acid	RARA	P10276	CHEMBL2055	0.053556	19 / 0

Palmitic Acid	RARB	P10826	CHEMBL2008	0.053556	135 / 0
Palmitic Acid	RARG	P13631	CHEMBL2003	0.053556	129 / 0
Carvone Hydrate	RASGRP3	Q8IV61	CHEMBL3638	0.116739	0 / 1
Daucosterol	RBP4	P02753	CHEMBL3100	0.074392	2 / 0
2- Dodecenal	RELA	Q04206	CHEMBL5533	0.071787	1 / 0
5Z-Dodecenyl Acetate	RET	P07949	CHEMBL2041	0.060425	37 / 0
Methyl caffeate	RNASEH1	O60930	CHEMBL5893	0.031227	1 / 0
Carvone Hydrate	RORA	P35398	CHEMBL5868	0.133391	0 / 2
Palmitic Acid	RORB	Q92753	CHEMBL3091268	0.053556	1 / 0
2α,19α-Dihydroxyursolic acid	RORC	P51449	CHEMBL1741186	0.128067	0 / 10
Cosmosiine	RPS6KA3	P51812	CHEMBL2345	0.118883	0 / 19
Rosmarinic acid ethyl ester	RPS6KB1	P23443	CHEMBL4501	0.115737	179 / 0
(+/-)-Eriodictyol	RXRA	P19793	CHEMBL2061	0.097875	210 / 0
Palmitic Acid	RXRB	P28702	CHEMBL1870	0.053556	69 / 0
Coleonolic acid	RXRG	P48443	CHEMBL2004	0.120226	20 / 0
Daucosterol	S1PR1	P21453	CHEMBL4333	0.074392	129 / 0
Coleonolic acid	S1PR2	O95136	CHEMBL2955	0.120226	20 / 0
Phytone	S1PR3	Q99500	CHEMBL3892	0.112042	101 / 0
Daucosterol	S1PR4	O95977	CHEMBL3230	0.074392	14 / 0
Daucosterol	S1PR5	Q9H228	CHEMBL2274	0.074392	3 / 0
Arachic acid	SAE1 UBA2	Q9UBE0 Q9UBT2	CHEMBL2095174	0.111502	0 / 1
Carvone Hydrate	SCD	O00767	CHEMBL5555	0.125076	0 / 1
5Z-Dodecenyl Acetate	SCN4A	P35499	CHEMBL2072	0.060425	8 / 0
Linalool	SCN5A	Q14524	CHEMBL1980	0.043919	3 / 0
5Z-Dodecenyl Acetate	SCN9A	Q15858	CHEMBL4296	0.060425	17 / 0
Rosmarinic acid	SELE	P16581	CHEMBL3890	0.10934	11 / 0
Rosmarinic acid	SELL	P14151	CHEMBL3161	0.10934	11 / 0
Rosmarinic acid	SELP	P16109	CHEMBL5378	0.10934	17 / 0
Carvone Hydrate	SERPINA6	P08185	CHEMBL2421	0.125076	0 / 19
Coleonolic acid	SERPINE1	P05121	CHEMBL3475	0.120226	12 / 0
Rosmarinic acid methyl ester	SFRP1	Q8N474	CHEMBL5517	0.113286	1 / 0
Rosmarinic acid methyl ester	SGK1	O00141	CHEMBL2343	0.113286	5 / 0
Carvone Hydrate	SHBG	P04278	CHEMBL3305	0.125076	0 / 33
(1R,2R,4R)-Trihydroxy-p-menthane	SHH	Q15465	CHEMBL5602	0.08937	0 / 10
Carvone Hydrate	SIGMAR1	Q99720	CHEMBL287	0.191772	18 / 4
Decyl acetate	SIRT2	Q8IXJ6	CHEMBL4462	0.031227	7 / 0
Palmitic Acid	SLC16A1	P53985	CHEMBL4360	0.053556	13 / 0
2- Dodecenal	SLC18A3	Q16572	CHEMBL4767	0.071787	1 / 0
Apigenin	SLC22A12	Q96S37	CHEMBL6120	0.116965	68 / 0
2α,19α-Dihydroxyursolic acid	SLC22A6	Q4U2R8	CHEMBL1641347	0.110612	2 / 2
CAS 1038743-23-5	SLC28A2	O43868	CHEMBL5780	0.055648	16 / 0
Miscanthoside	SLC28A3	Q9HAS3	CHEMBL5707	0.106543	1 / 1
Cosmosiine	SLC29A1	Q99808	CHEMBL1997	0.118883	2 / 0

CAS 1038743-23-5	SLC5A1	P13866	CHEMBL4979	0.055648	51 / 23
CAS 1038743-23-5	SLC5A2	P31639	CHEMBL3884	0.055648	102 / 30
Miscanthoside	SLC5A4	Q9NY91	CHEMBL1770047	0.106543	1 / 12
Caffeic acid	SLC6A2	P23975	CHEMBL222	0.071787	0 / 1
Carvone Hydrate	SLC6A3	Q01959	CHEMBL238	0.133391	77 / 2
2α,19α-Dihydroxyursolic acid	SLC6A4	P31645	CHEMBL228	0.110612	0 / 1
5Z-Dodecenyl Acetate	SLC9A1	P19634	CHEMBL2781	0.060425	31 / 0
Gallocatechol	SNCA	P37840	CHEMBL6152	0.101614	12 / 0
Myrtenol	SPHK1	Q9NYA1	CHEMBL4394	0.043919	0 / 2
2- Dodecenal	SPHK2	Q9NRA0	CHEMBL3023	0.071787	0 / 1
Gallic acid	SQLE	Q14534	CHEMBL3592	0.150098	1 / 0
Gallocatechol	SRC	P12931	CHEMBL267	0.101614	5 / 8
Carvone Hydrate	SRD5A1	P18405	CHEMBL1787	0.125076	39 / 0
Carvone Hydrate	SRD5A2	P31213	CHEMBL1856	0.125076	0 / 19
β-sitosterol	SREBF2	Q12772	CHEMBL1795166	0.17281	0 / 1
(+/-)-Eriodictyol	ST3GAL3	Q11203	CHEMBL3596076	0.097875	0 / 1
Apigenin	ST6GAL1	P15907	CHEMBL3596075	0.108771	0 / 2
Caffeic acid	STAT3	P40763	CHEMBL4026	0.071787	0 / 1
Caffeic acid	SYK	P43405	CHEMBL2599	0.071787	0 / 1
5Z-Dodecenyl Acetate	TAAR1	Q96RJ0	CHEMBL5857	0.060425	0 / 8
Phytone	TACR1	P25103	CHEMBL249	0.112042	152 / 0
Phytone	TAOK3	Q9H2K8	CHEMBL5701	0.112042	16 / 0
5Z-Dodecenyl Acetate	TAS2R31	P59538	CHEMBL2034804	0.060425	0 / 1
Decyl acetate	TBXA2R	P21731	CHEMBL2069	0.031227	96 / 0
Apigenin	TBXAS1	P24557	CHEMBL1835	0.141522	27 / 0
3,7-Dimethylocta-2,6-dienyl propanoate	TDO2	P48775	CHEMBL2140	0.042894	21 / 0
2α,19α-Dihydroxyursolic acid	TERT	O14746	CHEMBL2916	0.110612	1 / 3
Terpinyl Acetate	TGFBR1	P36897	CHEMBL4439	0.031227	2 / 0
2- Dodecenal	TGM2	P21980	CHEMBL2730	0.071787	18 / 0
Coleonolic acid	THRA	P10827	CHEMBL1860	0.120226	15 / 0
Coleonolic acid	THRB	P10828	CHEMBL1947	0.120226	18 / 0
Caffeic acid	TLR4	O00206	CHEMBL5255	0.071787	2 / 21
Geranyl acetate	TMIGD3	P0DMS9	CHEMBL3712907	0.031227	6 / 0
Carvone Hydrate	TNF	P01375	CHEMBL1825	0.125076	0 / 8
Rosmarinic acid methyl ester	TNKS	O95271	CHEMBL6164	0.113286	11 / 0
Rosmarinic acid methyl ester	TNKS2	Q9H2K2	CHEMBL6154	0.113286	30 / 0
	TNNC1	P63316			
Linalool	TNNT2	P45379	CHEMBL2095202	0.043919	2 / 0
	TNNI3	P19429			
Carvone Hydrate	TOP1	P11387	CHEMBL1781	0.125076	0 / 2
Oleanolic acid	TOP2A	P11388	CHEMBL1806	0.100634	1 / 2
Coleonolic acid	TP53	P04637	CHEMBL4096	0.120226	4 / 0
Caffeic acid	TPMT	P51580	CHEMBL2500	0.071787	4 / 0
3,7-Dimethylocta-2,6-dienyl propanoate	TRPA1	O75762	CHEMBL6007	0.042894	1 / 2

Phytone	TRPM8	Q7Z2W7	CHEMBL1075319	0.112042	1 / 0
beta-Caryophyllene	TRPV1	Q8NER1	CHEMBL4794	0.042894	0 / 2
Vinyl Amyl Carbinol	TRPV3	Q8NET8	CHEMBL5522	0.044308	1 / 0
Phytone	TSPO	P30536	CHEMBL5742	0.112042	214 / 0
Perillyl acetate	TTL	Q8NG68	CHEMBL5549	0.031227	0 / 12
3,4-Dihydroxy-5-methoxybenzaldehyde	TTR	P02766	CHEMBL3194	0.133391	3 / 5
Geranyl acetate	TUBB1	Q9H4B7	CHEMBL1915	0.031227	10 / 0
Vinyl Amyl Carbinol	TYK2	P29597	CHEMBL3553	0.023833	24 / 0
Icariside E4	TYMS	P04818	CHEMBL1952	0.103187	5 / 0
Protocatechualdehyde	TYR	P14679	CHEMBL1973	0.044308	0 / 1
Rotungenoside	TYRO3	Q06418	CHEMBL5314	0.035898	26 / 0
(1R,2R,4R)-Trihydroxy-p-menthane	UGT2B7	P16662	CHEMBL4370	0.08057	0 / 20
Perillyl acetate	USP10	Q14694	CHEMBL3407323	0.031227	1 / 0
Perillyl acetate	USP13	Q92995	CHEMBL3407324	0.031227	1 / 0
5Z-Dodecenyl Acetate	VCAM1	P19320	CHEMBL3735	0.060425	19 / 0
Amentoflavone	VCP	P55072	CHEMBL1075145	1	1 / 1
Arachic acid	VDR	P11473	CHEMBL1977	0.150982	0 / 2
Amentoflavone	VEGFA	P15692	CHEMBL1783	1	1 / 1
Rosmarinic acid ethyl ester	WEE1	P30291	CHEMBL5491	0.115737	54 / 0
Cosmosiine	XDH	P47989	CHEMBL1929	0.118883	3 / 19
Terpinyl Acetate	XPO1	O14980	CHEMBL5661	0.031227	2 / 0
Gallocatechol	YWHAG	P61981	CHEMBL1293296	0.101614	1 / 0

Table S3. Summary of ALl-Gene Associations. In DisGeNET database, 93 ALI-related genes were obtained.

Disease	Gene	UniProt	DSI_g	DPI_g	Score_gda	First_Ref	Last_Ref
Acute Lung Injury	VIP	P01282	0.437	0.846	0.5	1996	2010
Acute Lung Injury	CXCL2	P19875	0.513	0.885	0.5	2009	2016
Acute Lung Injury	PPARG	P37231	0.358	0.885	0.5	2006	2011
Acute Lung Injury	ICAM1	P05362	0.364	0.962	0.5	2007	2016
Acute Lung Injury	TGFB1	P01137	0.287	0.962	0.3	2005	2005
Acute Lung Injury	APOA1	P02647	0.429	0.885	0.3	2011	2011
Acute Lung Injury	TFF2	Q03403	0.61	0.538	0.3	2010	2010
Acute Lung Injury	FAS	P25445	0.372	0.923	0.3	2000	2000
Acute Lung Injury	TFF1	P04155	0.529	0.769	0.3	2010	2010
Acute Lung Injury	FASLG	P48023	0.43	0.885	0.3	2000	2000
Acute Lung Injury	SFTPD	P35247	0.5	0.846	0.3	2018	2018
Acute Lung Injury	IL17A	Q16552	0.324	0.923	0.3	2017	2017
Acute Lung Injury	ASCL1	P50553	0.552	0.692	0.3	2010	2010
Acute Lung Injury	MYLK	Q15746	0.51	0.846	0.3	2009	2009
Acute Lung Injury	NFE2L2	Q16236	0.357	0.885	0.3	2015	2015
Acute Lung Injury	TNFAIP6	P98066	0.582	0.731	0.3	2016	2016
Acute Lung Injury	TXN	P10599	0.447	0.885	0.3	2005	2005
Acute Lung Injury	EPHX1	P07099	0.5	0.846	0.3	2016	2016
Acute Lung Injury	EDN1	P05305	0.38	0.846	0.3	2006	2006
Acute Lung Injury	IL1RL1	Q01638	0.547	0.731	0.3	2011	2011
Acute Lung Injury	CAT	P04040	0.359	0.962	0.3	2004	2004
Acute Lung Injury	ACVR1	Q04771	0.535	0.731	0.3	2011	2011
Acute Lung Injury	IL1B	P01584	0.276	0.962	0.21	1996	2012
Acute Lung Injury	PLAU	P00749	0.425	0.923	0.21	2008	2013
Acute Lung Injury	BAX	Q07812	0.417	0.885	0.2	2010	2010
Acute Lung Injury	BCL2	P10415	0.291	0.885	0.2	2010	2010
Acute Lung Injury	CCL2	P13500	0.321	0.962	0.2	2012	2012
Acute Lung Injury	C3	P01024	0.445	0.885	0.2	2012	2012
Acute Lung Injury	FADD	Q13158	0.537	0.846	0.2	2013	2013
Acute Lung Injury	PTGS2	P35354	0.314	0.962	0.2	2009	2009
Acute Lung Injury	MFN1	Q8IWA4	0.644	0.577	0.2	2015	2015
Acute Lung Injury	IL13	P35225	0.386	0.846	0.2	2004	2004
Acute Lung Injury	PLA2G2A	P14555	0.488	0.808	0.2	2009	2009
Acute Lung Injury	AKAP12	Q02952	0.566	0.615	0.2	2013	2013
Acute Lung Injury	PECAM1	P16284	0.426	0.846	0.2	2012	2012
Acute Lung Injury	PAWR	Q96IZ0	0.542	0.846	0.2	2007	2007
Acute Lung Injury	CCL4	P13236;Q8NHW4	0.466	0.923	0.2	2000	2012
Acute Lung Injury	CXCL6	P80162	0.582	0.692	0.2	1995	1995
Acute Lung Injury	CAV1	Q03135	0.388	0.885	0.2	2014	2014
Acute Lung Injury	TNF	P01375	0.231	0.962	0.2	2000	2012
Acute Lung Injury	TLR4	O00206	0.321	0.962	0.2	2009	2009
Acute Lung Injury	SCGB1A1	P11684	0.546	0.808	0.2	2008	2008

Acute Lung Injury	TIMP2	P16035	0.425	0.846	0.2	1999	1999
Acute Lung Injury	THBD	P07204	0.457	0.769	0.2	2009	2009
Acute Lung Injury	VEGFA	P15692	0.266	0.923	0.2	2012	2012
Acute Lung Injury	MFN2	O95140	0.471	0.846	0.2	2015	2015
Acute Lung Injury	XDH	P47989	0.529	0.846	0.2	1998	1998
Acute Lung Injury	CASP3	P42574	0.351	0.923	0.2	2018	2018
Acute Lung Injury	CASP8	Q14790	0.404	0.923	0.2	2018	2018
Acute Lung Injury	SLPI	P03973	0.449	0.846	0.2	1999	2015
Acute Lung Injury	P2RY12	Q9H244	0.533	0.731	0.2	2011	2011
Acute Lung Injury	NOD2	Q9HC29	0.423	0.923	0.2	2012	2012
Acute Lung Injury	PAK1	Q13153	0.494	0.808	0.2	2013	2013
Acute Lung Injury	SERPINE1	P05121	0.359	0.885	0.2	2000	2015
Acute Lung Injury	FCGR1A	P12314	0.532	0.731	0.2	2009	2009
Acute Lung Injury	FCGR2A	P12318	0.48	0.769	0.2	2009	2009
Acute Lung Injury	FCGR2B	P31994	0.522	0.846	0.2	2009	2009
Acute Lung Injury	FGA	P02671	0.573	0.769	0.2	2012	2012
Acute Lung Injury	FLT1	P17948	0.419	0.846	0.2	2012	2012
Acute Lung Injury	GJA1	P17302	0.393	0.885	0.2	2013	2013
Acute Lung Injury	GOT1	P17174	0.601	0.692	0.2	2015	2015
Acute Lung Injury	GSR	P00390	0.494	0.846	0.2	1994	1994
Acute Lung Injury	F3	P13726	0.415	0.846	0.2	2012	2012
Acute Lung Injury	F2	P00734	0.415	0.885	0.2	2012	2012
Acute Lung Injury	PROCR	Q9UNN8	0.56	0.692	0.2	2009	2009
Acute Lung Injury	ADRA2A	P08913	0.578	0.615	0.2	2011	2011
Acute Lung Injury	CYP1A1	P04798	0.436	0.846	0.2	2006	2006
Acute Lung Injury	NQO1	P15559	0.434	0.885	0.2	2014	2016
Acute Lung Injury	AGER	Q15109	0.42	0.885	0.2	2013	2013
Acute Lung Injury	ELANE	P08246	0.447	0.846	0.2	2004	2008
Acute Lung Injury	EPHX2	P34913	0.568	0.654	0.2	2012	2012
Acute Lung Injury	ALB	P02768	0.317	0.962	0.2	2012	2012
Acute Lung Injury	HMGB1	P09429	0.368	0.923	0.2	2011	2013
Acute Lung Injury	HMOX1	P09601	0.381	0.923	0.2	2010	2011
Acute Lung Injury	OPA1	O60313	0.51	0.846	0.2	2015	2015
Acute Lung Injury	NOS2	P35228	0.364	0.923	0.2	2006	2006
Acute Lung Injury	MIR330		0.584	0.692	0.2	2014	2014
Acute Lung Injury	MMP9	P14780	0.305	0.923	0.2	2012	2012
Acute Lung Injury	MMP7	P09237	0.446	0.885	0.2	2011	2011
Acute Lung Injury	MMP2	P08253	0.333	0.923	0.2	2012	2012
Acute Lung Injury	MIR96		0.536	0.769	0.2	2014	2014
Acute Lung Injury	KDR	P35968	0.378	0.885	0.2	2012	2012
Acute Lung Injury	ITGB2	P05107	0.444	0.923	0.2	1992	1992
Acute Lung Injury	AQP5	P55064	0.552	0.769	0.2	2014	2014
Acute Lung Injury	IFNG	P01579	0.288	0.962	0.2	2012	2012
Acute Lung Injury	IL1RN	P18510	0.373	0.923	0.2	1996	2005

Acute Lung Injury	IL2	P60568	0.336	0.885	0.2	2012	2012
Acute Lung Injury	IL4	P05112	0.332	0.962	0.2	2014	2014
Acute Lung Injury	IL6	P05231	0.248	0.962	0.2	2009	2009
Acute Lung Injury	IL10	P22301	0.281	0.923	0.2	2013	2013
Acute Lung Injury	IL18	Q14116	0.365	0.923	0.2	2013	2013
Acute Lung Injury	ADAR	P55265	0.501	0.808	0.2	2009	2009
Acute Lung Injury	MST1R	Q04912	0.542	0.808	0.01	2006	2006
